# Hamstring Mechanics During Acceleration, Deceleration and Sidestep Cutting

**DOI:** 10.1111/sms.70257

**Published:** 2026-03-31

**Authors:** Nikolai Steventon‐Lorenzen, Emily Fitzwilliam, Anthony G. Schache, David Opar, Nirav Maniar

**Affiliations:** ^1^ School of Behavioural and Health Sciences Australian Catholic University Melbourne Australia; ^2^ Sports Performance, Recovery, Injury and New Technologies (SPRINT) Research Centre Australian Catholic University Melbourne Australia; ^3^ Centre for Sport Research in the Institute for Physical Activity and Nutrition, School of Exercise and Nutrition Sciences Deakin University Burwood Australia; ^4^ La Trobe Sport and Exercise Medicine Research Centre (LASEM) La Trobe University Melbourne Victoria Australia

**Keywords:** biomechanics, hamstring strain injury, musculoskeletal modeling, running

## Abstract

Hamstring strain injuries (HSI) are common in field‐based sports. Musculoskeletal modeling studies have been used to investigate hamstring mechanics during steady‐state running. Accelerative and decelerative running as well as change of direction actions, which are common mechanisms of HSI, have been largely overlooked. Therefore, the aim of the present study was to investigate the mechanics of the biarticular hamstrings during acceleration, deceleration, and sidestep cutting tasks. Three‐dimensional motion analysis, ground reaction force and electromyography data were collected for 20 recreationally active adults performing acceleration, deceleration and 45‐degree sidestep cutting. Musculoskeletal modeling was used to solve for musculotendinous (MTU) force, stretch, and work. Peak MTU force occurred during acceleration for biceps femoris long head (1.5 body weights (BW)) and semimembranosus (2.7 BW), whereas it occurred during deceleration for semitendinosus (0.7 BW). Peak stretch was highest in deceleration for all hamstrings (10.9% to 13.7%), followed by sidestep cutting (8.9% to 12.4%) then acceleration (5.5% to 10.2%). The greatest negative work performed by biceps femoris long head occurred during acceleration and sidestep cutting (−0.25 J·kg^−1^) but occurred during sidestep cutting for semimembranosus (−0.50 J·kg^−1^) and deceleration for semitendinosus (−0.12 J·kg^−1^). Acceleration, deceleration and sidestep cutting impose substantial demands on the hamstrings. Deceleration imposed the largest kinematic (i.e., stretch) demand on all hamstrings, whilst the task imposing the greatest kinetic demands (i.e., force and negative work) varied depending on the individual hamstring. These findings may help to inform HSI preventative and return‐to‐sport strategies.

## 1 Introduction

Hamstring strain injuries (HSIs) are common in popular field‐based sports [1] and have a high recurrence rate [2], resulting in substantial convalescence and associated financial burden [3]. Despite this, HSI rates have remained unchanged for the past 30 years [1]. This suggests that current preventative strategies may not adequately prepare the hamstrings for the mechanical demands required during common sporting tasks. To inform better interventions, a detailed understanding of hamstring mechanics during common sporting tasks is required.

Hamstring strain injuries often occur during high‐speed running, where the late swing phase is the most commonly identified period of injury [4, 5, 6, 7, 8]. To understand the contributing factors, studies have used 3D motion capture experiments with musculoskeletal modeling analysis to investigate hamstring mechanics during high‐speed running, yielding important insights for the field. For example, studies have consistently shown that the biarticular hamstrings perform negative work during the late swing phase, while simultaneously reaching their peak length and force production [9, 10, 11, 12, 13]. This suggests that conditioning the hamstrings to withstand these demands is critical for injury risk reduction and rehabilitative efforts. Moreover, such studies have consistently shown that the biceps femoris long head (BFLH) musculotendinous unit (MTU) experiences a greater peak stretch (i.e., length relative to anatomical position) compared to the semimembranosus (SM) and semitendinosus (ST) [11, 13, 14], which may explain why more than 80% of HSIs occur to this muscle [15, 16]. Despite these insights, a notable limitation of the research completed to date is that steady‐state running conditions have primarily been investigated.

Recent studies have shown that HSIs in field‐based sports commonly occur during acceleration, deceleration and change of direction efforts [4, 5, 6]. There have been limited musculoskeletal modeling investigations of acceleration [17, 18], and none focusing on the swing phase of decelerative or change of direction efforts. A comprehensive understanding of the mechanical demands (i.e., MTU force, stretch, and work) of acceleration, deceleration and change of direction, and the differences between them, may provide valuable insight into how to reduce the risk of HSI or inform the appropriate reintroduction of tasks in return to sports programming. Therefore, the aim of the current study was to quantify the mechanics of the biarticular hamstrings during acceleration, deceleration, and sidestep cutting efforts, and to compare how these demands differ between each task.

## 2 Methods

### 2.1 Participants

Motion capture experiments were performed on 20 recreationally active adults (*n* = 10 males, *n* = 10 females; age, 25 ± 4 years; height, 1.73 ± 0.09 m; mass, 70 ± 10 kg). The sample size was originally determined for a previous investigation of joint mechanics [19], but we verified adequate power for the present objectives (Supporting Information S1). All participants were required to be free from any musculoskeletal injury or injury history which may have limited their ability to engage in high intensity running tasks via the Exercise and Sports Science Adult Pre‐Exercise Screening System. Participants gave their informed consent, and ethical approval was granted by the Australian Catholic University Human Research Ethics Committee (approval number: 2019‐351H).

### 2.2 Data Collection

Sixty‐one retroreflective markers were placed on key anatomical landmarks across the trunk, lower and upper limbs of each participant (Supporting Information S2—Table S1). Three‐dimensional marker trajectories were collected via a 10‐camera motion analysis system (VICON, Oxford Metrics Ltd., Oxford, United Kingdom) sampling at 200hz. Ground reaction force data was collected via 2 in‐series ground embedded force plates (Advanced Mechanical Technology Inc., Watertown, MA, USA) sampling at 1000 Hz. Muscle activity from 11 lower limb muscles (gluteus maximus, BFLH, ST, medial gastrocnemius, gluteus medius, adductor longus, rectus femoris, vastus lateralis, soleus, tibialis anterior, peroneus longus) was collected via electromyography (EMG) sensors (Cometa Miniwave Infinity, Cometa Scientific Ltd., Milan, Italy) sampling at 1000 Hz for each participant's right leg. We designated the right leg as the test leg for all participants for ease of analysis. Sensor placement was performed in line with Surface Electromyography for the Non‐Invasive Assessment of Muscle guidelines [20], except for the adductor longus sensor which does not have guidelines. In this instance, the sensor was placed at 50% of the length of the muscle belly while the participant was positioned in supine with simultaneous hip external rotation and knee flexion to allow for palpation of the adductor longus muscle belly.

Data collection commenced with recording a static trial with the participant assuming a neutral stance pose in the middle of the capture volume. Each participant then completed a standardized progressive warm‐up, after which they performed the dynamic tasks of interest: maximal effort acceleration, deceleration, and 45‐degree sidestep cut. For all tasks, participants commenced each trial at their own initiation from a staggered, stationary starting position. For accelerations, participants were asked to accelerate forwards at their maximum capacity. For sidestep cuts, participants were asked to accelerate forwards and perform a pre‐planned 45‐degree sidestep cut (guided by tape on the floor) once they reached the designated zone within the capture volume. For decelerations, participants began each trial like the acceleration trials but responded to a light signal triggered by a timing gate system (Smartspeed, VALD Performance, Brisbane, Australia). If the lights turned green, participants continued to maximally accelerate and the trial was discarded. If the lights turned red, participants were required to decelerate (i.e., come to a complete stop) as quickly as possible. Participants' starting positions were adjusted by an investigator, such that their right leg landed on the ground embedded force plates during their second or third step in their natural gait pattern. Note that for decelerations and sidestep cutting, this meant that the analyzed swing phase corresponded to the one just before the first decelerative foot contact (for deceleration) or change of direction (for sidestep cutting, often referred to as the “final foot contact”). This process ensured that the same step number was analyzed for all trials across all tasks, facilitating more valid comparisons. Trials were repeated until three successful trials of each task were collected for each participant (i.e., nine trials total). A successful trial required that the participant's right foot clearly landed within the boundaries of one of the force plates while completing the designated task.

### 2.3 Data Processing

Marker trajectories and ground reaction forces were filtered using a zero‐lag, 4th order Butterworth filter with a cut‐off frequency of 15Hz [21]. EMG data were corrected for offset, high‐pass filtered (20 Hz), full‐wave rectified, and low‐pass filtered (6 Hz) using a zero‐lag, 4th order Butterworth filter, then normalized to the peak amplitude obtained across the nine trials.

### 2.4 Musculoskeletal Modeling

A 37 degree‐of‐freedom model, with 98 lower limb and trunk MTU actuators and 14 ideal torque actuators in the upper limbs [22, 23, 24] was used to perform musculoskeletal simulations in OpenSim (v4.5) [25]. As per our previous work [19], we locked DOFs related to the wrist and metatarsophalangeal joints, leaving 31 degrees‐of‐freedom in the model. To reduce computation time, we removed MTU actuators on the left limb and trunk and replaced them with ideal torque actuators. We made minor modifications to muscle paths when they demonstrated physiologically infeasible solutions due to wrap‐surface errors [26].

Simulations were generated by: (1) scaling a generic model to the anthropometry of each participant taken from the static trial; (2) applying a global optimization inverse kinematics algorithm to solve for joint angles; (3) applying inverse dynamics to solve for net joint moments; (4) using a static optimization algorithm to solve for muscle forces by minimizing the sum of squared muscle activations; and (5) solving for time dependent changes in MTU length via a muscle analysis. We computed MTU stretch (often referred to as strain), which expresses the change in MTU length as a percentage of its length during anatomical position. Lengthening velocity of each MTU was calculated as the first derivative of MTU length with respect to time and multiplied by muscle forces to compute MTU power. To compute MTU work, MTU power was integrated with respect to time. This musculoskeletal modeling pipeline has been used to estimate muscle forces and muscle kinematics in previous studies analyzing high‐speed running [9].

### 2.5 Outcome Variables

Outcome variables were extracted for the biarticular hamstring muscles only as per previous research [10, 12, 14]. The primary variables of interest were MTU force, stretch and work, owing to their direct relevance to injury [27]. Secondary outcome variables, MTU velocity and power, were also visualized to support interpretation but were not the primary focus of our analysis. Forces were normalized to body weight (BW) while power and work were normalized to body mass. For time‐varying quantities (i.e., force, power, stretch, and velocity), we computed ensemble mean and SD curves for visualization for a complete stride cycle from toe‐off (determined by the peak positive vertical toe velocity [28]) to the subsequent ipsilateral toe‐off, as per other work investigating hamstring mechanics [14]. For subsequent statistical analysis, we extracted the peak force, power absorption, stretch and lengthening velocity, as well as the total negative work, during the swing phase. We also calculated approach velocity as the average anterior center of mass velocity during the swing phase.

### 2.6 Validation and Verification

The results of our biomechanical simulations were validated and verified using best practice recommendations [29]. Joint kinematics and moments had temporal agreement with previous studies on the stance phase of accelerative [30] and decelerative [31] running and sidestep cutting [32] (Supporting Information S3—Figure S2 to S4). Simulated muscle forces had reasonable temporal agreement with experimental EMG, and the simulated muscle moments agreed with moments derived from inverse dynamics (Supporting Information S4—Figure S7 to S12). We also visually inspected individual time curves (Supporting Information S5—Figure S13 to S16) of all relevant outcome data and excluded any trials that had clear errors. This process resulted in the exclusion of 9 trials before statistical analysis. Since the missing data pattern was minor (5%) and deemed completely random, no adjustment to our statistical analysis was performed (see Supporting Information S6 for further details of our missing data).

### 2.7 Statistical Analysis

Statistical analysis was performed in R Statistical Computing Software Version 4.4 (R Foundation for Statistical Computing, Vienna, Austria) using the Tidyverse [33], lme4 [34], and emmeans [35] packages. Linear mixed models were used to determine if any of the outcome variables significantly differed between tasks (fixed effect) while accounting for participant ID (random intercepts). Random slopes for participant were not modeled due to convergence issues. Given that varying approach velocities between tasks could confound our comparisons, it was also included as a covariate, such that marginal estimates and comparisons were adjusted for approach velocity. Assumptions for the statistical models were verified via visual inspection of residual and qqplots, and where significant main effects were found for task, post hoc pairwise contrasts were conducted using Tukey's method (*p* < 0.05).

## 3 Results

### 3.1 Descriptives

**FIGURE 1:**
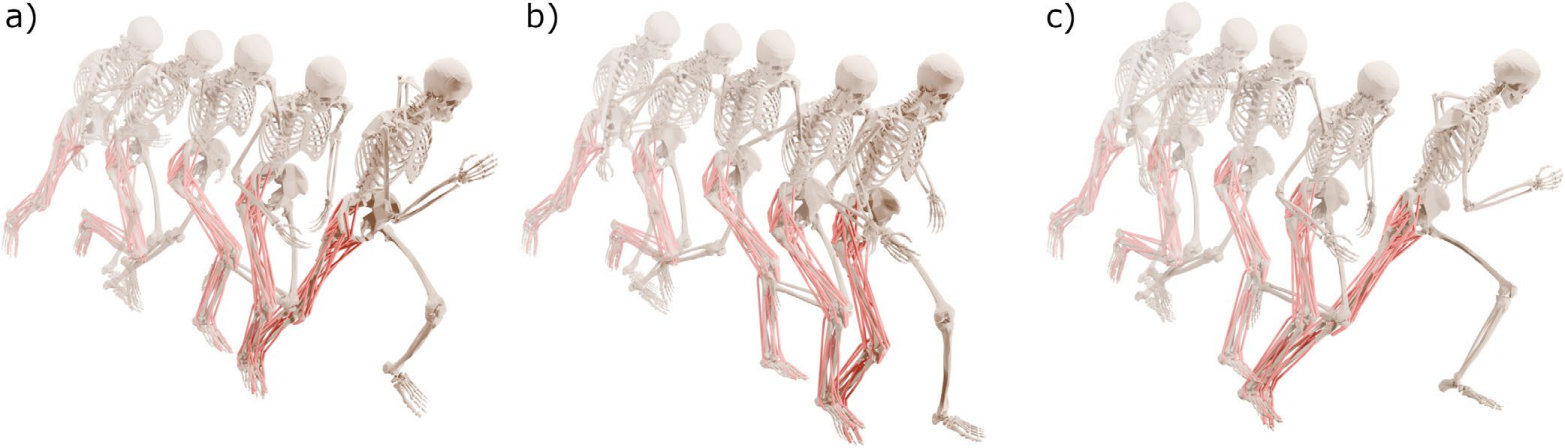
Visual representation of the average kinematics of acceleration, deceleration, and sidestep cutting from 0% (toe‐off) to 100% (ipsilateral toe‐off).

A visualization of the average kinematics of each task is provided in Figure 1. The mean ± SD approach velocities for acceleration, deceleration and sidestep cutting were 3.6 ± 0.4 m·s^−1^, 3.5 ± 0.5 m·s ^−1^, and 3.3 ± 0.4 m·s ^−1^, respectively. The swing phase of acceleration, deceleration and sidestep cutting accounted for 63% ± 3%, 59% ± 6% and 60% ± 3% of the stride cycle, respectively. Marginal means and 95% CI for each outcome variable are described in Table 1, whereas all statistical results are provided in Supporting Information S7—Table S2 to S4.

**TABLE 1:** Marginal means and 95% CI of peak hamstring mechanical variables during acceleration, deceleration and 45‐degree sidestep cutting.

Muscle	Marginal mean and 95% CI		
	Acceleration	Sidestep	Deceleration
Force (BW)			
Biceps femoris long head	1.5 [1.3 to 1.6]	1.2 [1.0 to 1.4]	1.2 [1.0 to 1.4]
Semimembranosus	2.7 [2.4 to 3.0]	2.3 [2.0 to 2.6]	1.9 [1.6 to 2.2]
Semitendinosus	0.4 [0.3 to 0.5]	0.5 [0.5 to 0.6]	0.7 [0.7 to 0.8]
Power absorption (W·kg^−1^)			
Biceps femoris long head	−4.8 [−5.7 to −4.0]	−3.8 [−4.7 to −3.0]	−3.6 [−4.4 to −2.7]
Semimembranosus	−6.0 [−7.3 to −4.7]	−8.6 [−10.0 to −7.3]	−8.7 [−10.0 to −7.4]
Semitendinosus	−0.7 [−1.0 to −0.3]	−1.6 [−2.0 to −1.3]	−2.3 [−2.7 to −2.0]
Negative work (J·kg^−1^)			
Biceps femoris long head	−0.25 [−0.29 to −0.21]	−0.25 [−0.29 to −0.21]	−0.21 [−0.25 to −0.17]
Semimembranosus	−0.33 [−0.38 to −0.28]	−0.50 [−0.56 to −0.45]	−0.43 [−0.48 to −0.37]
Semitendinosus	−0.05 [−0.06 to −0.04]	−0.10 [−0.11 to −0.09]	−0.12 [−0.13 to −0.11]
Stretch (%)			
Biceps femoris long head	10.2 [9.2 to 11.2]	12.4 [11.4 to 13.5]	13.7 [12.7 to 14.7]
Semimembranosus	5.5 [4.5 to 6.6]	8.9 [7.8 to 10.0]	10.9 [9.9 to 12.0]
Semitendinosus	6.4 [5.2 to 7.6]	10.2 [9.0 to 11.4]	12.4 [11.2 to 13.6]
Lengthening velocity (m·s^−1^)			
Biceps femoris long head	0.65 [0.61 to 0.70]	0.66 [0.61 to 0.70]	0.68 [0.64 to 0.73]
Semimembranosus	0.61 [0.56 to 0.65]	0.67 [0.63 to 0.72]	0.76 [0.71 to 0.80]
Semitendinosus	0.72 [0.68 to 0.77]	0.78 [0.73 to 0.84]	0.86 [0.81 to 0.91]

*Note:* Marginal estimates were adjusted to the mean approach velocity (3.48 m·s^−1^).

Abbreviations: CI, confidence interval; BW, bodyweight.

### 3.2 Force

**FIGURE 2:**
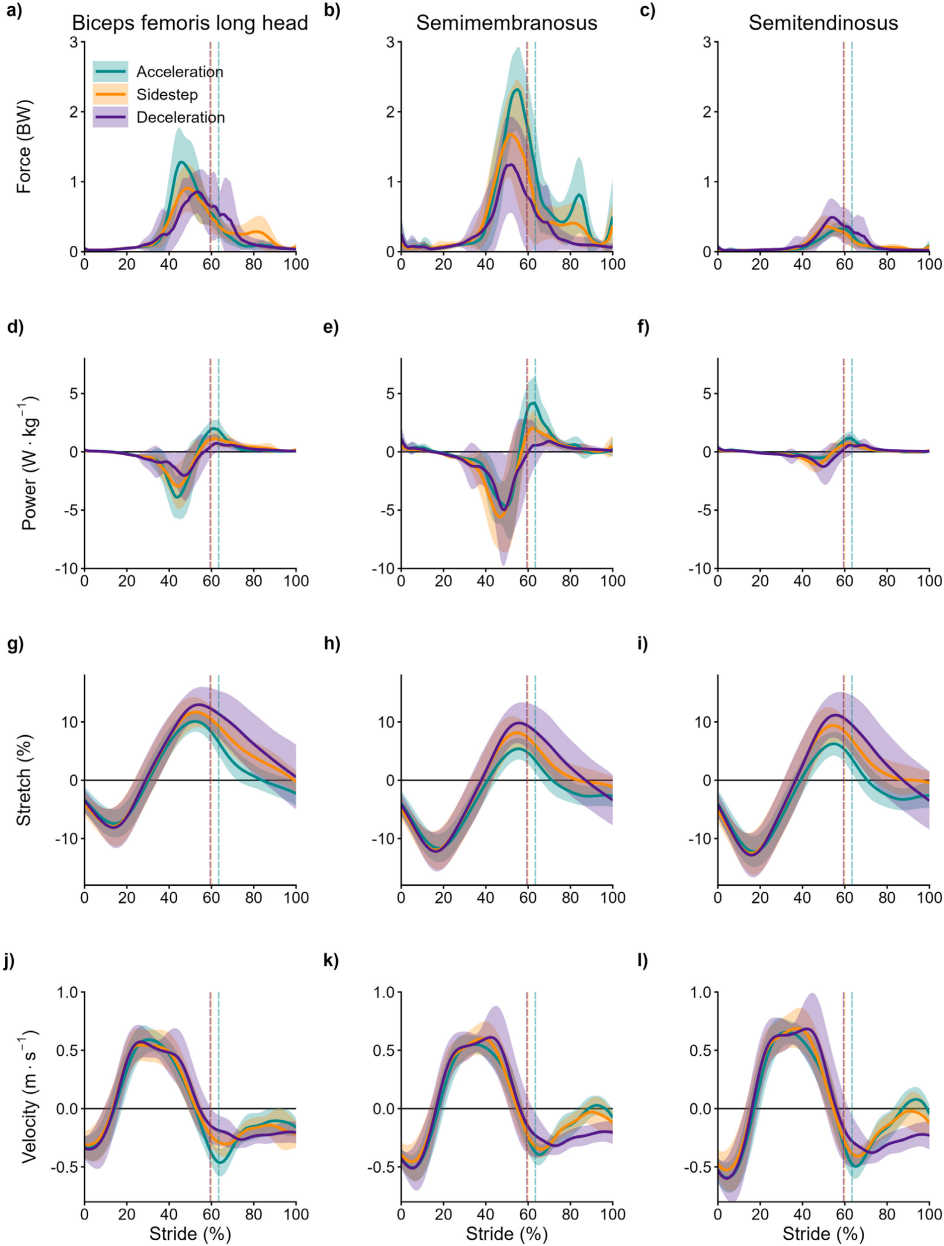
Mean (line) and SD (shaded) of the musculotendinous force (first row), power (second row), stretch (third row), and lengthening velocity (fourth row) for the biceps femoris long head (first column), semimembranosus (second column), and semitendinosus (third column) across the stride cycle (toe‐off to toe‐off) of acceleration (green), deceleration (purple), and 45‐degree sidestep cutting (orange). The vertical dashed line indicates the beginning of the stance phase for each task. The late swing phase (i.e., peak hip flexion) for acceleration, deceleration and sidestep cutting commenced at 44% ± 3%, 42% ± 5%, and 42% ± 4% of the stride cycle, respectively. Force data was normalized to bodyweight (BW). Power was normalized to body mass (W·kg^−1^), where positive values indicate generation and negative values indicate absorption. Stretch represents the length of the musculotendinous unit relative to anatomical position, where positive values indicate lengths greater than anatomical position. Positive values for velocity indicate lengthening.

Temporal patterns in muscle forces displayed only minor qualitative differences between muscles or tasks (Figure 2). In general, force production gradually increased during the swing phase, peaking during late swing for all three hamstrings, regardless of task. The peak force of the BFLH was significantly higher for acceleration (1.5 BW, 95% CI = 1.3 to 1.6 BW) compared to deceleration (1.2 BW, 95% CI = 1.0 to 1.4 BW, *p* < 0.001) and sidestep cutting (1.2 BW, 95% CI = 1.0 to 1.4 BW, *p* < 0.001), whilst differences between deceleration and sidestep cutting were not statistically significant (*p* = 0.970). For the SM, all tasks were significantly different from each other, with the greatest peak force observed during acceleration (2.7 BW, 95% CI = 2.4 to 3.0 BW) followed by sidestep cutting (2.3 BW, 95% CI = 2.0 to 2.6 BW, *p* = 0.007) and deceleration (1.9 BW, 95% CI = 1.6 to 2.2 BW, *p* < 0.001 for acceleration, *p* = 0.001 for sidestep). Peak force for ST was also significantly different between all tasks; however, the greatest force was observed in deceleration (0.7 BW, 95% CI = 0.7 to 0.8 BW) followed by sidestep cutting (0.5 BW, 95% CI = 0.5 to 0.6 BW, *p* < 0.001) and acceleration (0.4 BW, 95% CI = 0.3 to 0.5 BW, *p* < 0.001 for both comparisons).

### 3.3 Power and Work

**FIGURE 3:**
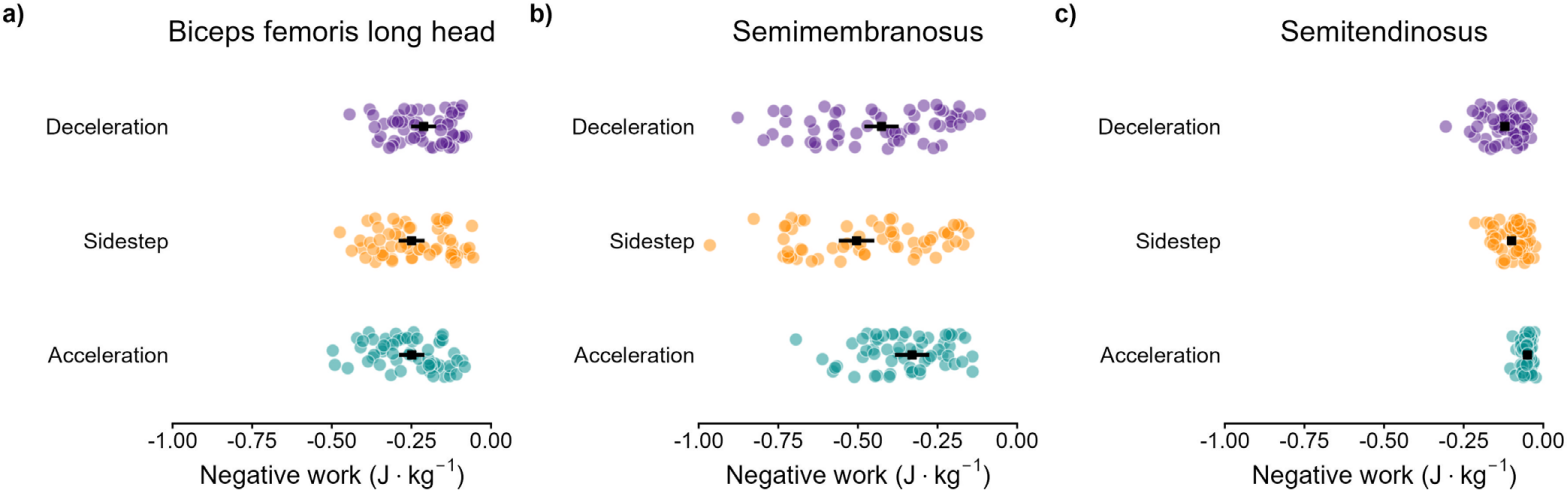
The body mass normalized negative work for the biceps femoris long head (first column), semimembranosus (second column), and semitendinosus (third column) during the swing phase of acceleration (green), deceleration (purple), and 45‐degree sidestep cutting (orange).

The temporal pattern of MTU power was similar for all hamstrings, characterized by a gradual increase in power absorption over the swing phase, followed by a brief period of power generation near (and after) foot strike for all tasks (Figure 2). For BFLH, the negative work during swing was not significantly different for acceleration and sidestep cutting (−0.25 J·kg^−1^, 95% CI = −0.29 to −0.21 J·kg^−1^ for both tasks, *p* = 1.000), whilst both were significantly greater than deceleration (−0.21 J·kg^−1^, 95% CI = −0.25 to −0.17 J·kg^−1^, *p* = 0.010 for acceleration, *p* = 0.021 for sidestep cutting; Figure 3). For the SM, the greatest negative work was observed during sidestep cutting (−0.50 J·kg^−1^, 95% CI = −0.56 to −0.45 J·kg^−1^), followed by deceleration (−0.43 J·kg^−1^, 95% CI = −0.48 to −0.37 J·kg^−1^, *p* = 0.010) then acceleration (−0.33 J·kg^−1^, 95% CI = −0.38 to −0.28 J·kg^−1^, *p* < 0.001 for sidestep, *p* = 0.001 for deceleration). For the ST, the greatest negative work was observed during deceleration (−0.12 J·kg^−1^, 95% CI = −0.13 to −0.11 J·kg^−1^), followed by sidestep cutting (−0.10 J·kg^−1^, 95% CI = −0.11 to −0.09 J·kg^−1^, *p* = 0.005) then acceleration (−0.05 J·kg^−1^, 95% CI = −0.06 to −0.04 J·kg^−1^, *p* < 0.001 for both comparisons). Comparisons of peak power absorption can be found in Table 1.

### 3.4 Stretch and Velocity

All hamstrings underwent a period of shortening for the initial portion of the swing phase, followed by rapid lengthening to a peak stretch during late swing, before briefly shortening before foot strike and then continuing to shorten throughout stance (Figure 2). Peak stretch was significantly different between all tasks, for all muscles (*p* < 0.001 for all comparisons). For all muscles, peak stretch was highest for deceleration (BFLH: 13.7%, 95% CI = 12.7 to 14.7%; SM: 10.9%, 95% CI = 9.9 to 12.0%; ST: 12.4%, 95% CI = 11.2 to 13.6%) followed by sidestep cutting (BFLH: 12.4%, 95% CI = 11.4 to 13.5%; SM: 8.9%, 95% CI = 7.8 to 10.0%; ST: 10.2%, 95% CI = 9.0 to 11.4%) then acceleration (BFLH: 10.2%, 95% CI = 9.2 to 11.2%; SM: 5.5%, 95% CI = 4.5 to 6.6%; ST: 6.4%, 95% CI = 5.2 to 7.6%). Comparisons of peak lengthening velocity can be found in Table 1.

## 4 Discussion

The goal of the present study was to compare the mechanical demands of the biarticular hamstrings during the swing phase of maximal acceleration, deceleration, and sidestep cutting. Our main findings were: (1) peak force for BFLH and SM was highest when accelerating whereas for ST it was highest when decelerating; (2) peak stretch was greatest when decelerating for all hamstrings, followed by sidestep cutting and acceleration; and (3) negative work differed between tasks, where the rank order varied depending on the muscle. These findings have direct implications for preventative and rehabilitative interventions aiming to condition the hamstrings for sports involving acceleration, deceleration and sidestep cutting actions.

Hamstring mechanics during the swing phase of acceleration, deceleration and sidestep cutting actions has received little attention to date. Although this limits direct comparison of our estimates to previous research, several key observations provide confidence in our findings. Firstly, the large stretch and loading (i.e., force, negative work) for each hamstring during late swing is consistent with previous studies in high‐speed running [9, 10, 11, 12]. Muscle‐specific trends for each hamstring also show consistency with previous work, such as the greatest peak stretch in the BFLH compared to the other hamstrings, and largest kinetic estimates in the SM compared to the other hamstrings [9, 10, 11, 12, 14]. The magnitudes of our estimates are also reasonable compared with previous work, especially when considering the different demands of top speed running.

### 4.1 Force

Our results showed that peak hamstring muscle force significantly varied across tasks (Table 1). Although previous research has shown peak hamstring forces to increase with increasing steady‐state running velocities [11], our approach‐velocity adjusted analysis shows that peak hamstring muscle forces are also influenced by intent to accelerate and change direction. This finding suggests that protocols designed to progressively increase mechanical demands on the hamstrings need to consider varying task type, in addition to increments in velocity. Importantly, the influence of task varied between the hamstring muscles, with the BFLH and SM experiencing peak force during acceleration and the ST during deceleration, suggesting that loading progressions may be muscle specific.

Although comparing our muscle force estimates to other studies should be interpreted with caution (due to different sample characteristics and modeling approaches), such comparisons can speculatively yield clinical insight. For example, our peak BFLH force estimates across tasks (~1.2 to 1.5 BW, on average) are less than previous estimates of maximal sprinting (8.95 m·s ^−1^, 2.7 BW) [9] and in resistance training exercises like single‐leg deadlifts (1.5 BW), isometric roman chair holds (1.8 BW), and the Nordic hamstring exercise (2.3 BW) [36]. This might suggest that resistance training exercise can impose muscle force demands that exceed the tasks observed in our study. Whilst similar observations can be made for the SM, our observed peak force for the ST (~0.4 to 0.7 BW, on average) was similar or greater than that previously observed in the single‐leg deadlift (0.4 BW), isometric roman chair holds (0.6 BW), and sprinting (8.95 m·s ^−1^, 0.6 BW), but not the Nordic hamstring exercise (2.4 BW) [9, 36]. This indicates that deceleration is particularly demanding on the ST, perhaps more so than some commonly prescribed resistance training exercises other than the Nordic hamstring exercise.

### 4.2 Stretch

Analysis of hamstring MTU stretch has been largely limited to steady‐state running [9, 10, 11, 12, 13, 14], with more recent work examining acceleration [17, 18]. These studies suggest that accelerative running imposes greater peak stretch compared to velocity‐matched steady‐state running [17] or maximum velocity sprinting [18]. Our study builds on these findings, showing that peak MTU stretch during deceleration and sidestep cutting were significantly greater than acceleration for all hamstring muscles (Table 1). Indeed, the average peak stretch observed in deceleration (BFLH: 13.7%, SM: 10.9%, ST: 12.4%) and sidestep cutting (BFLH: 12.4%, SM: 8.9%, ST: 10.2%) are also greater than that previously observed in acceleration [18] (BFLH: 9.0%, SM: 6.1%, ST: 6.1%) and steady‐state sprinting (9.0 m·s ^−1^, BFLH: 11.5%, SM: 9.4%, ST: 8.3%) and lower velocity running [14]. The larger MTU stretch observed during deceleration is perhaps unsurprising, given the differences in kinematics (Figure S2), where individuals adopted greater hip flexion and knee extension angles than in other tasks (Figure 1). This action increased the anterior displacement of the foot relative to the center of mass before foot contact, potentially to increase the braking ground reaction forces and subsequently reduce the forward center of mass momentum [37]. A similar (albeit less extreme) kinematic strategy may also explain the higher MTU stretch in sidestep cutting compared to accelerative and steady‐state running, as greater braking forces (Figure S2) are required before redirection [32].

### 4.3 Negative Work

The negative work estimated in our analysis represents the accumulated force generation under MTU lengthening conditions, which may reflect accumulated eccentric loading, which has been associated with muscle damage [38]. As many field‐based sports require repetitive bouts of acceleration, deceleration, and sidestep cutting [39], the accumulation of muscle damage may have implications for HSI risk. Our results show that the negative work imposed by each task depends on the specific hamstring muscle. For example, decelerative efforts imposed the most negative work for the ST, while sidestep cutting imposed greater negative work for the SM (Table 1). The BFLH, however, has a similar level of negative work during sidestep cutting and acceleration. Such knowledge may therefore inform load monitoring strategies to decrease the risk of injury during training and match play.

### 4.4 Limitations

Our sample of 20 recreationally active healthy adults means that our findings cannot be generalized to other populations groups, such as higher‐level athletes or those with a previous HSI. The muscle forces derived using static optimization in this study cannot be directly validated against in vivo muscle forces. The temporal muscle force patterns were shown to have reasonable qualitative agreement with experimental EMG profiles giving us some confidence in our approach (Supporting Information S4). However, it is important to recognize that alternative muscle force estimation methods or subject‐specific models (e.g., informed by magnetic resonance imaging) may yield different muscle force patterns and magnitudes. Our analysis describes hamstring mechanics at the MTU level with a rigid tendon. In reality, the hamstring tendons are compliant structures, which can decouple length changes in the MTU from those in the muscle fibres [27]. It is therefore important to recognize that our findings should not be confused with fiber mechanics (e.g., stretch at the MTU level is not necessarily equal to stretch at the muscle fiber level). To facilitate valid comparisons between acceleration, deceleration and sidestep cutting, our analysis was restricted to swing phase of an equivalent “matched” step. For deceleration and sidestep cutting, this corresponded to the first decelerative step (for deceleration) and the change of direction step (for sidestep cutting). Our results should therefore be interpreted as the “step‐specific” mechanical demands, in consideration of the constraints imposed (e.g., approach velocity, cutting angles, etc.). It should be considered that different swing phases (e.g., the step after the sidestep cut or another accelerative/decelerative step), different change of direction angles, approach velocities and acceleration/deceleration intensities could yield alternative findings. Future studies may aim to explore the influence of these additional variables. Finally, our statistical analysis considered each mechanical variable separately. In reality, no single variable alone defines HSI risk, it is more likely that variables such as force, stretch, and negative work interact in a complex manner to contribute to HSI susceptibility.

## 5 Conclusion

The current study investigated the mechanics of the biarticular hamstrings during acceleration, deceleration, and sidestep cutting tasks. We found that deceleration imposed the greatest peak stretch on all hamstrings, followed by sidestep cutting and acceleration. Kinetic demands (i.e., forces and negative work) imposed by different tasks, however, varied depending on the muscle. The greatest peak force was observed during acceleration for the BFLH and SM, but during deceleration for the ST. The BFLH experienced the greatest negative work during acceleration and sidestep cutting, whereas the SM and ST experienced the greatest negative work during sidestep cutting and deceleration, respectively. These muscle‐specific variations in task demands may be helpful for guiding hamstring injury prevention and rehabilitation interventions.

## 6 Perspective

Previous modeling studies investigating hamstring mechanics have largely focused on linear running at various steady‐state speeds [9, 10, 11, 12, 13, 14], demonstrating muscle‐specific differences in hamstring mechanics and how these mechanics differ with changing running speed. Given that HSIs also occur during acceleration, deceleration and change of direction actions [4, 5, 6], this study advances the field's knowledge of hamstring mechanics for these fundamental athletic tasks. Our findings showed that hamstring mechanical loading significantly differs between tasks, suggesting that interventions aiming to prepare the hamstrings for the demands of sport (e.g., injury risk reduction and rehabilitation programs) need to consider varying accelerative and direction change requirements, in addition to speed of execution.

## Author Contributions

Nikolai Steventon‐Lorenzen: Methodology, Software, Validation, Formal analysis, Investigation, Data curation, Writing – Original draft; Emily Fitzwilliam: Investigation, Data curation, Writing – Review and Editing; Anthony Schache: Conceptualization, Methodology, Writing – Review and Editing; David Opar: Conceptualization, Methodology, Writing – Review and Editing, Supervision; Nirav Maniar: Conceptualization, Methodology, Software, Validation, Formal analysis, Investigation, Data curation, Writing – Review and Editing, Visualization, Project administration, Funding acquisition, Supervision.

## Funding

This work was supported by Australian Catholic University. Consumables were funded by the Faculty of Health Sciences Funding Support for Honors and Masters by Coursework with Minor Thesis scheme, provided by Australian Catholic University.

## Ethics Statement

Ethical approval was granted by the Australian Catholic University Human Research Ethics Committee (approval number: 2019‐351H).

## Consent

All participants provided written informed consent.

## Conflicts of Interest

Prof David Opar is listed as a co‐inventor on a patent (PCT/AU2012/001041.2012), filed by the Queensland University of Technology (QUT), for a field‐testing device of eccentric hamstring strength, which is now known commercially as the NordBord. Prof Opar has received revenue distributions from QUT based on revenue that QUT has generated through the commercialization of his intellectual property. Prof Opar is a minority shareholder in Vald Performance Pty Ltd., the company responsible for commercialization of the NordBord and the Smart Speed Timing gates, of which the latter were used in the current manuscript. Prof Opar has received research funding from Vald Performance, for work unrelated to the current manuscript. Prof Opar was previously the Chair of the Vald Performance Research Committee, a role that was unpaid. Prof Opar has family members who are minor shareholders and/or employees of Vald Performance.

## Supporting information


**Figure S1:** Power estimate (point) and 95% CI (error bars) for a stretch difference of 1% in the biceps femoris long head between tasks. As shown, a sample size of *n* = 20 was adequate to reach 80% power (gray dashed line).
**Table S1:** A description of retroreflective marker placement.
**Figure S2:** Mean (line) and SD (shaded area) joint angles for the stride cycle (toe‐off to toe‐off) of acceleration (green), 45‐degree sidestep cutting (orange) and deceleration (purple). Note that the stride cycle corresponds to the final foot contact before change of direction (for sidestep cutting) and the first decelerative step (for deceleration). Positive values indicate hip flexion, hip adduction, hip internal rotation, knee flexion and ankle dorsi flexion, for each subplot.
**Figure S3:** Mean (line) and SD (shaded area) body‐mass normalized joint moments for the stride cycle (toe‐off to toe‐off) of acceleration (green), 45‐degree sidestep cutting (orange) and deceleration (purple). Note that the stride cycle corresponds to the final foot contact before change of direction (for sidestep cutting) and the first decelerative step (for deceleration). Positive values indicate hip flexion, hip adduction, hip internal rotation, knee flexion and ankle dorsi flexion, for each subplot.
**Figure S4:** Mean (line) and SD (shaded area) joint angular velocities for the stride cycle (toe‐off to toe‐off) of acceleration (green), 45‐degree sidestep cutting (orange) and deceleration (purple). Note that the stride cycle corresponds to the final foot contact before change of direction (for sidestep cutting) and the first decelerative step (for deceleration). Positive values indicate hip flexion, hip adduction, hip internal rotation, knee flexion and ankle dorsi flexion, for each subplot.
**Figure S5:** Mean (line) and SD (shaded area) ground reaction forces for the stance phase of acceleration (green), 45‐degree sidestep cutting (orange) and deceleration (purple). Note that the stance phase corresponds to the final foot contact before change of direction (for sidestep cutting) and the first decelerative step (for deceleration). Positive values indicate anterior, vertical, and medial (i.e., toward the left for right‐foot contact), for each subplot.
**Figure S6:** Mean (line) and SD (shaded area) center of mass velocity for the stride cycle of acceleration (green), 45‐degree sidestep cutting (orange) and deceleration (purple). Note that the stride cycle corresponds to the final foot contact before change of direction (for sidestep cutting) and the first decelerative step (for deceleration). Positive values indicate anterior, vertical, and medial (i.e., toward the left for right‐foot contact), for each subplot. The vertical dashed lines indicate the beginning of the stance phase for each task.
**Figure S7:** Mean (line) and SD (shaded area) normalized electromyography (EMG, blue) and model‐derived muscle force (gray) for the stride cycle (toe‐off to toe‐off) for acceleration. Note that normalization was performed across all trials (including sidestep cutting and deceleration).
**Figure S8:** Mean (line) and SD (shaded area) normalized electromyography (EMG, blue) and model‐derived muscle force (gray) for the stride cycle (toe‐off to toe‐off) for 45‐degree sidestep cutting. Note that normalization was performed across all trials (including acceleration and deceleration).
**Figure S9:** Mean (line) and SD (shaded area) normalized electromyography (EMG, blue) and model‐derived muscle force (gray) for the stride cycle (toe‐off to toe‐off) for deceleration. Note that normalization was performed across all trials (including sidestep cutting and acceleration).
**Figure S10:** Mean (line) and SD (shaded area) body‐mass normalized joint moments from inverse dynamics (blue) and model‐derived muscle moment (gray) for the stride cycle (toe‐off to toe‐off) for acceleration.
**Figure S11:** Mean (line) and SD (shaded area) body‐mass normalized joint moments from inverse dynamics (blue) and model‐derived muscle moment (gray) for the stride cycle (toe‐off to toe‐off) for 45‐degree sidestep cutting.
**Figure S12:** Mean (line) and SD (shaded area) body‐mass normalized joint moments from inverse dynamics (blue) and model‐derived muscle moment (gray) for the stride cycle (toe‐off to toe‐off) for deceleration.
**Figure S13:** Mean (thick line) and individual (faded lines) of the bodyweight (BW) normalized musculotendinous forces for the biceps femoris long head (top row), semimembranosus (second row) and semitendinosus (bottom row) for the stride cycle (toe‐off to toe‐eff) of acceleration (green, first column), deceleration (purple, second column), and 45‐degree sidestep cutting (orange, third column). Note that the stride cycle corresponds to the final foot contact before change of direction (for sidestep cutting) and the first decelerative step (for deceleration).
**Figure S14:** Mean (thick line) and individual (faded lines) of the body mass normalized musculotendinous power for the biceps femoris long head (top row), semimembranosus (second row) and semitendinosus (bottom row) for the stride cycle (toe‐off to toe‐eff) of acceleration (green, first column), deceleration (purple, second column), and 45‐degree sidestep cutting (orange, third column). Note that the stride cycle corresponds to the final foot contact before change of direction (for sidestep cutting) and the first decelerative step (for deceleration). Positive values indicate power generation.
**Figure S15:** Mean (thick line) and individual (faded lines) of the musculotendinous (MTU) stretch for the biceps femoris long head (top row), semimembranosus (second row) and semitendinosus (bottom row) for the stride cycle (toe‐off to toe‐eff) of acceleration (green, first column), deceleration (purple, second column), and 45‐degree sidestep cutting (orange, third column). Note that the stride cycle corresponds to the final foot contact before change of direction (for sidestep cutting) and the first decelerative step (for deceleration). Positive values indicate MTU lengths greater than anatomical position.
**Figure S16:** Mean (thick line) and individual (faded lines) of the musculotendinous (MTU) velocity for the biceps femoris long head (top row), semimembranosus (second row) and semitendinosus (bottom row) for the stride cycle (toe‐off to toe‐eff) of acceleration (green, first column), deceleration (purple, second column), and 45‐degree sidestep cutting (orange, third column). Note that the stride cycle corresponds to the final foot contact before change of direction (for sidestep cutting) and the first decelerative step (for deceleration). Positive values indicate MTU lengthening.
**Figure S17:** Visualization of missing (dark blue) and present (light blue) data for each trial (x‐axis) and participant (y‐axis) for data included in our statistical analysis and summary figures. Note that electromyography data had additional missing data (technical failures for two participants), but this was not included in this visualization as it was not related to our key outcomes.
**Table S2:** Marginal means and 95% CI confidence interval for secondary outcomes in the stance phase of acceleration, deceleration and sidestep cutting.
**Table S3:** Pairwise contrasts for linear mixed models of the swing phase for acceleration, deceleration and sidestep cutting.
**Table S4:** Pairwise contrasts for linear mixed models of the stance phase for acceleration, deceleration and sidestep cutting.

## Data Availability

The data that support the findings of this study are available from the corresponding author upon reasonable request, subject to the approval of the Australian Catholic University Human Research Ethics Committee.
