# Supplementary material for: Hamstring Mechanics During Acceleration, Deceleration and Sidestep Cutting
Source: Scand J Med Sci Sports. 2026 Mar 31;36(4):e70257. doi: 10.1111/sms.70257 (PMC13039759; doi:10.1111/sms.70257)
Supplement: Supplementary file 1 — Figure S1: Power estimate (point) and 95% CI (error bars) for a stretch difference of 1% in the biceps femoris long head between tasks. As shown, a sample size of n = 20 was adequate to reach 80% power (gray dashed line). Table S1: A description of retroreflective marker placement. Figure S2: Mean (line) and SD (shaded area) joint angles for the stride cycle (toe‐off to toe‐off) of acceleration (green), 45‐degree sidestep cutting (orange) and deceleration (purple). Note that the stride cycle corresponds to the final foot contact before change of direction (for sidestep cutting) and the first decelerative step (for deceleration). Positive values indicate hip flexion, hip adduction, hip internal rotation, knee flexion and ankle dorsi flexion, for each subplot. Figure S3: Mean (line) and SD (shaded area) body‐mass normalized joint moments for the stride cycle (toe‐off to toe‐off) of acceleration (green), 45‐degree sidestep cutting (orange) and deceleration (purple). Note that the stride cycle corresponds to the final foot contact before change of direction (for sidestep cutting) and the first decelerative step (for deceleration). Positive values indicate hip flexion, hip adduction, hip internal rotation, knee flexion and ankle dorsi flexion, for each subplot. Figure S4: Mean (line) and SD (shaded area) joint angular velocities for the stride cycle (toe‐off to toe‐off) of acceleration (green), 45‐degree sidestep cutting (orange) and deceleration (purple). Note that the stride cycle corresponds to the final foot contact before change of direction (for sidestep cutting) and the first decelerative step (for deceleration). Positive values indicate hip flexion, hip adduction, hip internal rotation, knee flexion and ankle dorsi flexion, for each subplot. Figure S5: Mean (line) and SD (shaded area) ground reaction forces for the stance phase of acceleration (green), 45‐degree sidestep cutting (orange) and deceleration (purple). Note that the stance phase corresponds to the fin [file SMS-36-e70257-s001.docx]

**Title**

Hamstring mechanics during acceleration, deceleration and sidestep cutting

**Authors**

Nikolai Steventon-Lorenzen ^a, b^, nikolai.steventonlorenzen@myacu.edu.au, ORCID: 0009-0007-2385-6468

Emily Fitzwilliam ^a, b, c^, emily.fitzwilliam@deakin.edu.au, ORCID: 0009-0007-6813-2586

Anthony G Schache ^d^, a.schache@latrobe.edu.au, ORCID: 0000-0002-1041-5213

David Opar ^a, b^, david.opar@acu.edu.au, ORCID: 0000-0002-8354-6353

Nirav Maniar ^a, b^, nirav.maniar@acu.edu.au, ORCID: 0000-0002-6180-6003

*Affiliations*

^a^ School of Behavioural and Health Sciences, Australian Catholic University, Melbourne, Australia

^b^ Sports Performance, Recovery, Injury and New Technologies (SPRINT) Research Centre, Australian Catholic University, Melbourne, Australia

^c^ Centre for Sport Research in the Institute for Physical Activity and Nutrition, School of Exercise and Nutrition Sciences, Deakin University, Burwood, Australia

^d^ La Trobe Sport and Exercise Medicine Research Centre (LASEM), La Trobe University, Melbourne, Victoria, Australia.

**Corresponding author**

Nirav Maniar

Email: [nirav.maniar@acu.edu.au](mailto:nirav.maniar@acu.edu.au)

Supplementary Material S1: Sample size estimation

## Background

Our original sample size of n=20 was selected based on a prior publication, which focused on comparing joint mechanics during acceleration and deceleration. To verify adequate power for the present study, we used a simulation approach via the lme4^1^ and simr^2^ packages in the R programming language^3^.

## Metric

We chose musculotendinous (MTU) stretch as the primary outcome of interest for sample size estimation for the following reasons.

1. stretch magnitude has a clear relationship with mechanical damage^4^
2. stretch is less sensitive to modelling assumptions than kinetic measures (e.g., force)
3. hamstring MTU stretch has been reported extensively for high intensity running^5–8^, is a common mechanism of hamstring strain injury ^9–11^, thus giving insight into potentially clinically relevant stretch magnitudes

## Statistical model

An initial linear mixed model was created using data for the biceps femoris long head. Although any hamstring muscle could have been selected, we chose this muscle as it is the most commonly injured hamstring. The model formula was:

$$\begin{matrix} \mathrm{stretch}_{i} & \sim N\left( \mu,\sigma^{2} \right) \\ \mu& =\alpha_{j\left[ i \right]}+\beta_{1}\left( \mathrm{task}_{\mathrm{cut}} \right)+\beta_{2}\left( \mathrm{task}_{\mathrm{decel}} \right)+\beta_{3}\left( \mathrm{velocity}_{approach} \right) \\ \alpha_{j} & \sim N\left( \mu_{\alpha_{j}},\sigma_{\alpha_{j}}^{2} \right)\text{, for participant j = 1,}\ldots\text{,J} \end{matrix}$$

Where:

- $\mathrm{stretch}_{i}$is the peak MTU stretch
- $\alpha_{j\left[ i \right]}$ is the random intercept, which varies for each participant ID level
- $task$ is the fixed effect representing the 3 different task (acceleration, deceleration and sidestep)
- $\beta_{1}\left( \mathrm{task}_{\mathrm{cut}} \right)$ is the coefficient for the sidestep cutting task, representing the change in peak MTU stretch relative to the intercept (noting that the intercept is designated as the task occurring first alphabetically, which was acceleration). A similar interpretation can be made for $\beta_{2}\left( \mathrm{task}_{\mathrm{decel}} \right)$.
- $\beta_{3}\left( \mathrm{velocity}_{approach} \right)$ is the approach velocity, computed as the average forward centre of mass velocity over the swing period

Random slopes for participant were not modeled due to convergence issues.

Next, we used the simr package^2^ to change the effect size (i.e., difference in peak stretch) for the first $\beta$ term (sidestep cutting) to 1%, relative to the model’s intercept (i.e., the peak stretch during the acceleration). A stretch difference of 1% was selected because prior work has shown the biceps femoris long head to experience a peak stretch of 1-3% more than the other long hamstrings^5,7,8^, yet accounts for over 80% of hamstring strain injuries ^12,13^.

Power was calculated for sample sizes from 5 to 20 (at intervals of 5 participants), with 5000 simulations per sample size (i.e., a total of 20000 simulations).


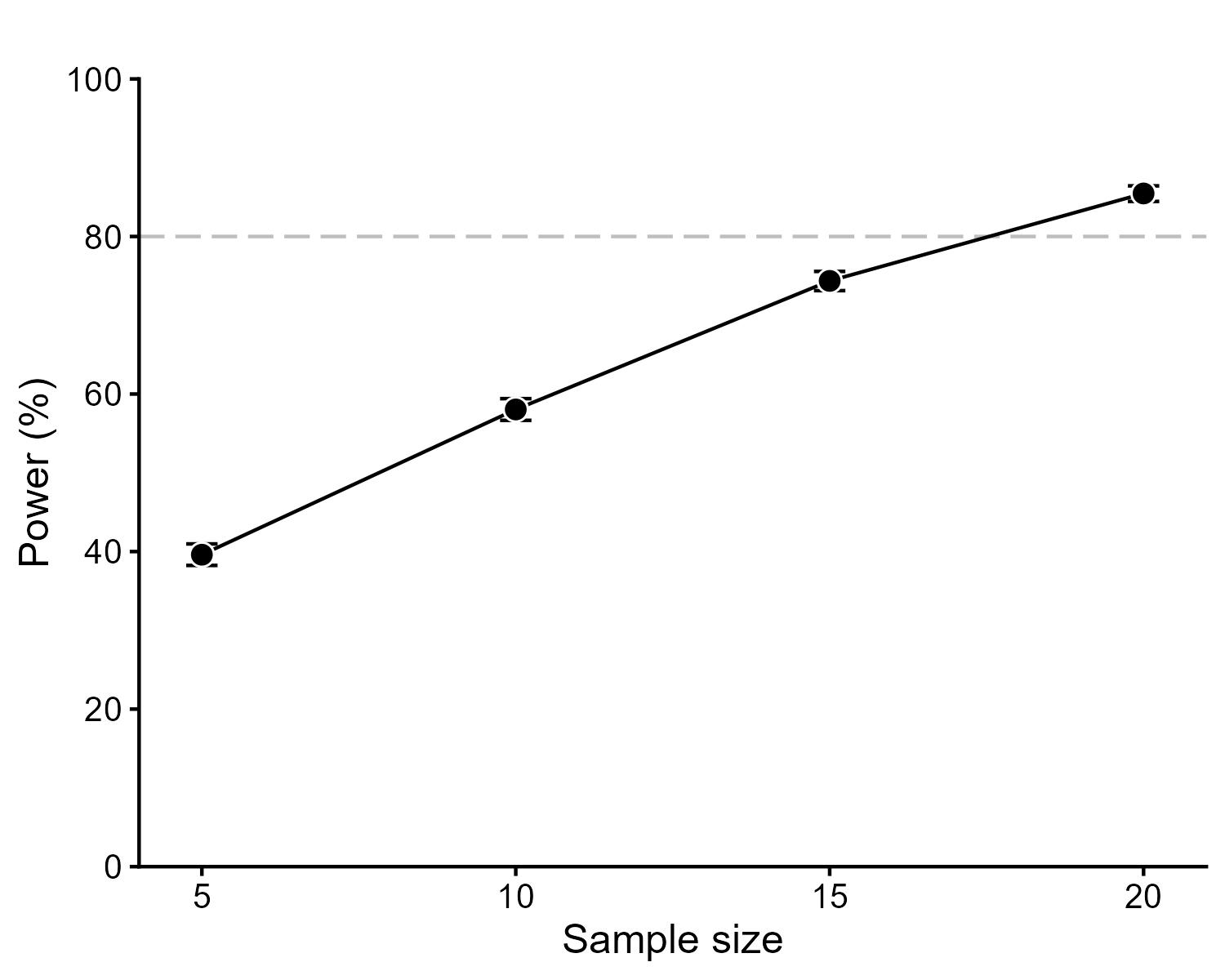


Supplementary Figure S1. Power estimate (point) and 95%CI (error bars) for a stretch difference of 1% in the biceps femoris long head between tasks. As shown, a sample size of n=20 was adequate to reach 80% power (grey dashed line).

## References

1. Bates, D., Mächler, M., Bolker, B. & Walker, S. Fitting Linear Mixed-Effects Models Using lme4. *2015* **67**, 48 (2015).

2. Green, P. & MacLeod, C. J. SIMR: an R package for power analysis of generalized linear mixed models by simulation. *Methods Ecol. Evol.* **7**, 493–498 (2016).

3. R Development Core Team. R: A language and environment for statistical computing. R Foundation for Statistical Computing (2010).

4. Brooks, S. V., Zerba, E. & Faulkner, J. A. Injury to muscle fibres after single stretches of passive and maximally stimulated muscles in mice. *J. Physiol.* **488 ( Pt 2)**, 459–469 (1995).

5. Schache, A. G., Dorn, T. W., Wrigley, T. V., Brown, N. A. & Pandy, M. G. Stretch and activation of the human biarticular hamstrings across a range of running speeds. *Eur. J. Appl. Physiol.* **113**, 2813–2828 (2013).

6. Schache, A. G., Dorn, T. W., Blanch, P. D., Brown, N. A. & Pandy, M. G. Mechanics of the human hamstring muscles during sprinting. *Med. Sci. Sports Exerc.* **44**, 647–58 (2012).

7. Chumanov, E. S., Heiderscheit, B. C. & Thelen, D. G. The effect of speed and influence of individual muscles on hamstring mechanics during the swing phase of sprinting. *J. Biomech.* **40**, 3555–3562 (2007).

8. Chumanov, E. S., Heiderscheit, B. C. & Thelen, D. G. Hamstring musculotendon dynamics during stance and swing phases of high speed running. *Med. Sci. Sports Exerc.* **43**, 525 (2011).

9. Jokela, A. *et al.* Mechanisms of Hamstring Injury in Professional Soccer Players: Video Analysis and Magnetic Resonance Imaging Findings. *Clin. J. Sport Med. Off. J. Can. Acad. Sport Med.* **33**, 217–224 (2023).

10. Kerin, F. *et al.* Its not all about sprinting: mechanisms of acute hamstring strain injuries in professional male rugby union-a systematic visual video analysis. *Br. J. Sports Med.* **56**, 608–615 (2022).

11. Gronwald, T. *et al.* Hamstring injury patterns in professional male football (soccer): a systematic video analysis of 52 cases. *Br. J. Sports Med.* **56**, 165–171 (2022).

12. Breed, R., Opar, D., Maniar, N. & Pizzari, T. Epidemiology of Hamstring Strain Injuries in Elite Male Australian Football Players: An Analysis of 773 Injuries Over 7 Seasons. *JOSPT Open* **2**, 289–296 (2024).

13. Koulouris, G., Connell, D. A., Brukner, P. & Schneider-Kolsky, M. Magnetic resonance imaging parameters for assessing risk of recurrent hamstring injuries in elite athletes. *Am J Sports Med* **35**, 1500–1506 (2007).

Supplementary Material S2: Marker set details

Supplementary Table S1. A description of retroreflective marker placement.

| Marker name | Description |
| --- | --- |
| *Torso* | |
| R_Acromion | Right acromion process of the scapula |
| L_Acromion | Left acromion process of the scapula |
| C7 | The most prominent aspect of the C7 vertebrae |
| Sternum | Inferior to the jugular notch of the manubrium |
| MidT | The midpoint between the C7 and PSIS markers |
| *Humerus (right)* | |
| R_Elbow_Lat | Right lateral epicondyle of the humerus |
| R_Elbow_Med | Right medial epicondyle of the humerus |
| *Forearm (right)* | |
| R_Radius | Right styloid process of the radius |
| R_Ulna | Right head of the ulna |
| *Humerus (left)* | |
| L_Elbow_Lat | Left lateral epicondyle of the humerus |
| L_Elbow_Med | Left medial epicondyle of the humerus |
| *Forearm (left)* | |
| L_Radius | Left styloid process of the radius |
| L_Ulna | Left head of the ulna |
| *Pelvis* | |
| R_ASIS | Right anterior superior iliac spine |
| L_ASIS | Left anterior superior iliac spine |
| R_PSIS | Right posterior superior iliac spine |
| L_PSIS | Left posterior superior iliac spine |
| *Femur (right)* | |
| R_Thigh_Post_Sup | Lateral thigh (cluster marker) |
| R_Thigh_Ant_Sup | Lateral thigh (cluster marker) |
| R_Thigh_Ant_Inf | Lateral thigh (cluster marker) |
| R_Knee_Lat | Lateral epicondyle of the femur |
| R_Knee_Med | Medial epicondyle of the femur |
| R_Thigh_Post_Inf | Lateral thigh (cluster marker) |
| *Shank (right)* | |
| R_Shank_Post_Sup | Lateral shank (cluster marker) |
| R_Shank_Ant_Sup | Lateral shank (cluster marker) |
| R_Ankle_Lat | Lateral malleolus |
| R_Ankle_Med | Medial malleolus |
| R_Shank_Post_Inf | Lateral shank (cluster marker) |
| R_Shank_Ant_Inf | Lateral shank (cluster marker) |
| *Foot (right)* | |
| R_Heel_Inf | Inferior aspect of the calcaneal tuberosity |
| R_MTP1 | Head of the 1st metatarsal |
| R_MTP5 | Head of the 5th metatarsal |
| R_Midfoot_Med | Navicular tuberosity |
| R_Midfoot_Lat | Base of 5th metatarsal |
| R_Toe | First distal phalanx of the hallux |
| R_Heel_Lat | Lateral process of the calcaneal tuberosity |
| R_Heel_Med | Medial process of the calcaneal tuberosity |
| R_MTP2 | Head of the 2nd metatarsal |
| R_Midfoot_Int | Midpoint of Midfoot_Med and Midfoot_Lat |
| R_Heel_Sup | Superior aspect of the calcaneal tuberosity |
| *Femur (left)* | |
| L_Thigh_Post_Sup | Lateral thigh (cluster marker) |
| L_Thigh_Ant_Sup | Lateral thigh (cluster marker) |
| L_Thigh_Post_Inf | Lateral thigh (cluster marker) |
| L_Knee_Lat | Lateral epicondyle of the femur |
| L_Knee_Med | Medial epicondyle of the femur |
| L_Thigh_Ant_Inf | Lateral thigh (cluster marker) |
| *Shank (left)* | |
| L_Shank_Ant_Sup | Lateral shank (cluster marker) |
| L_Shank_Ant_Inf | Lateral shank (cluster marker) |
| L_Ankle_Lat | Lateral malleolus |
| L_Ankle_Med | Medial malleolus |
| L_Shank_Post_Sup | Lateral shank (cluster marker) |
| L_Shank_Post_Inf | Lateral shank (cluster marker) |
| *Foot (left)* | |
| L_Heel_Inf | Inferior aspect of the calcaneal tuberosity |
| L_MTP1 | Head of the 1st metatarsal |
| L_MTP5 | Head of the 5th metatarsal |
| L_Midfoot_Med | Navicular tuberosity |
| L_Midfoot_Lat | Base of 5th metatarsal |
| L_Toe | First distal phalanx of the hallux |
| L_Heel_Lat | Lateral process of the calcaneal tuberosity |
| L_Heel_Med | Medial process of the calcaneal tuberosity |
| L_MTP2 | Head of the 2nd metatarsal |
| L_Midfoot_Int | Midpoint of Midfoot_Med and Midfoot_Lat |
| L_Heel_Sup | Superior aspect of the calcaneal tuberosity |

Supplementary Material S3: Supplementary data

This supplementary data file contains data supplementary to our main outcome data, necessary for validation and verification of our results. This includes ensemble mean and SD curves of the following variables:

- Lower limb joint angles
- Lower limb joint moments
- Lower limb joint angular velocity
- Ground reaction forces
- Centre of mass velocity


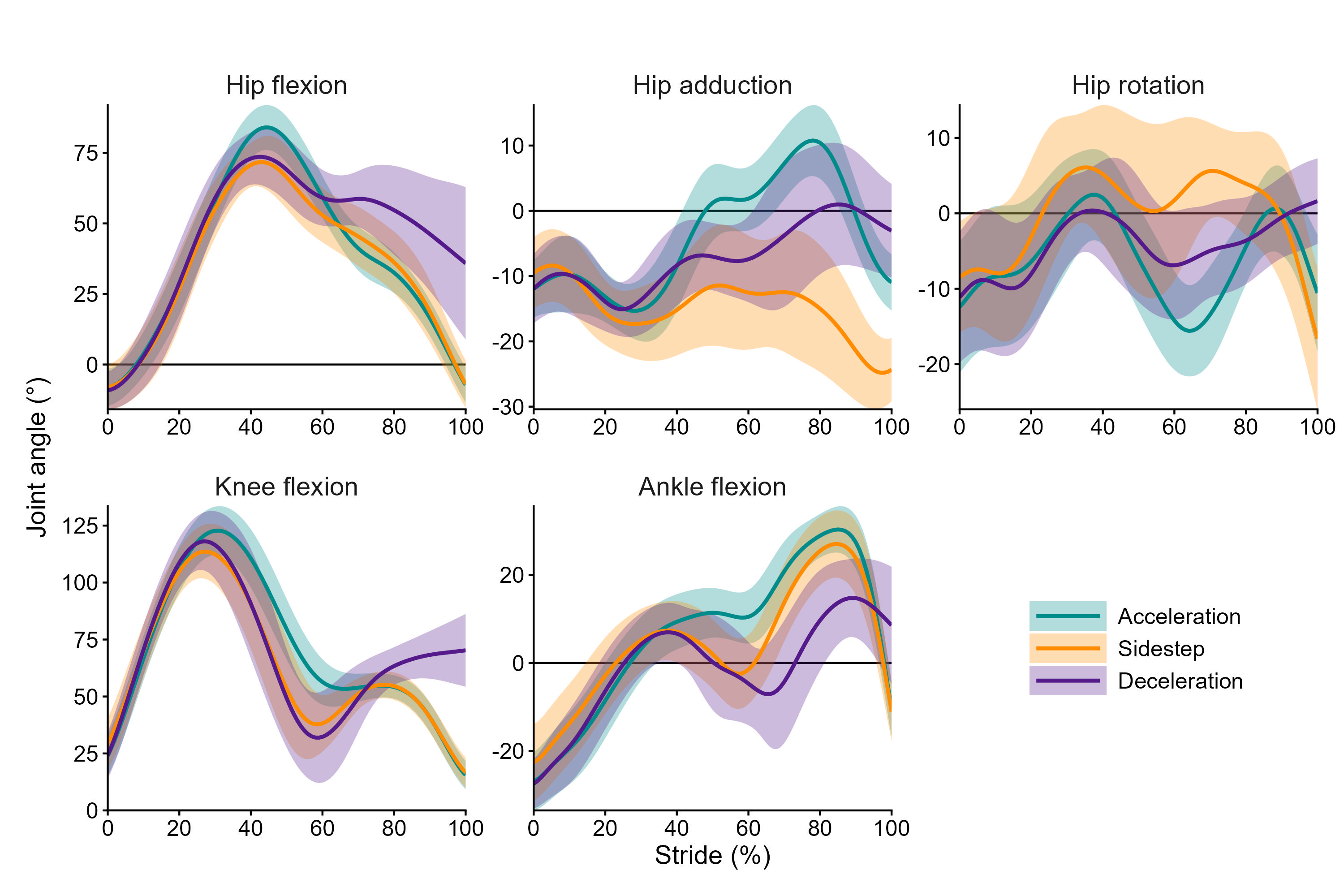


Supplementary Figure S2. Mean (line) and SD (shaded area) joint angles for the stride cycle (toe-off to toe-off) of acceleration (green), 45-degree sidestep cutting (orange) and deceleration (purple). Note that the stride cycle corresponds to the final foot contact prior to change of direction (for sidestep cutting) and the first decelerative step (for deceleration). Positive values indicate hip flexion, hip adduction, hip internal rotation, knee flexion and ankle dorsi flexion, for each subplot.


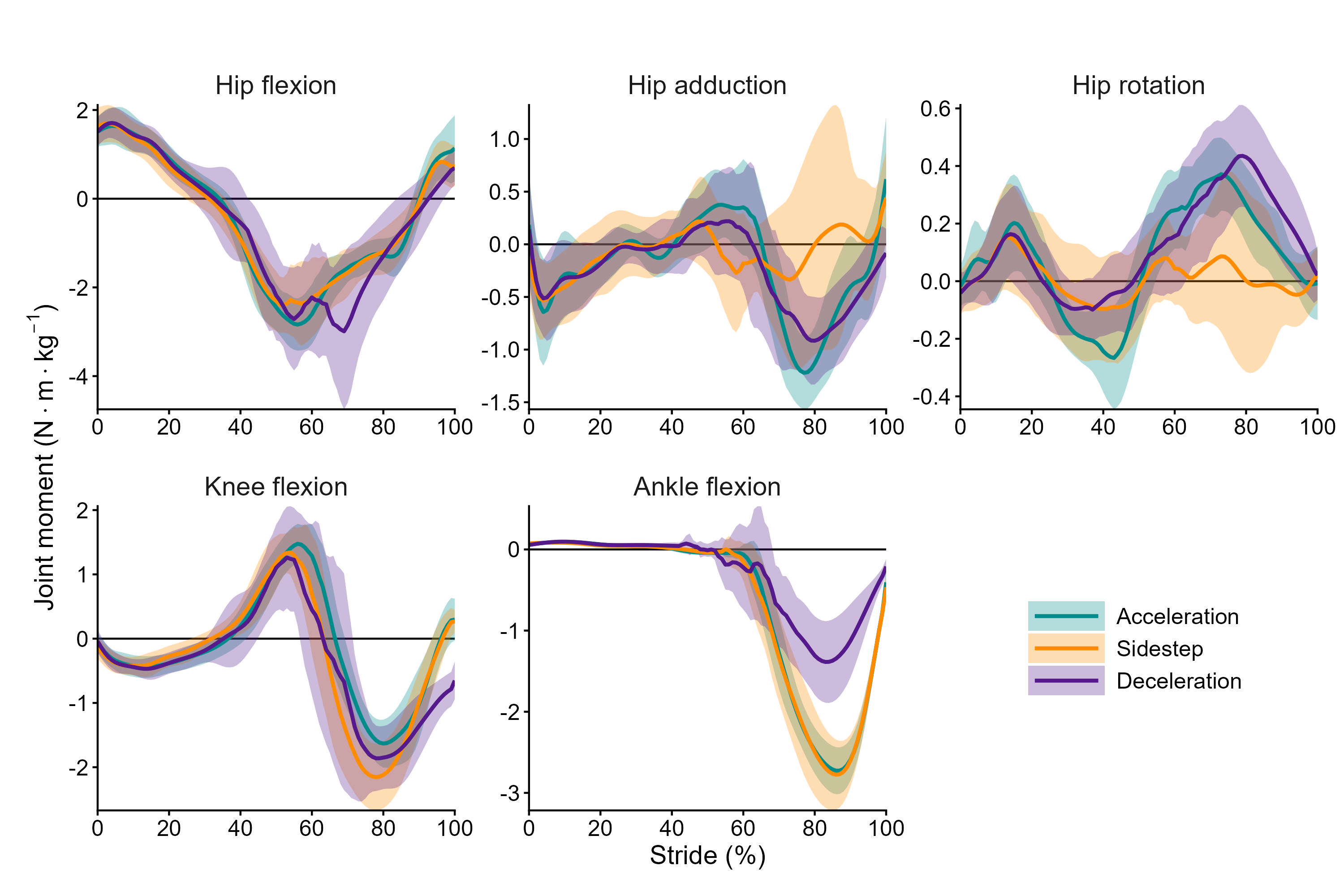


Supplementary Figure S3. Mean (line) and SD (shaded area) body-mass normalised joint moments for the stride cycle (toe-off to toe-off) of acceleration (green), 45-degree sidestep cutting (orange) and deceleration (purple). Note that the stride cycle corresponds to the final foot contact prior to change of direction (for sidestep cutting) and the first decelerative step (for deceleration). Positive values indicate hip flexion, hip adduction, hip internal rotation, knee flexion and ankle dorsi flexion, for each subplot.


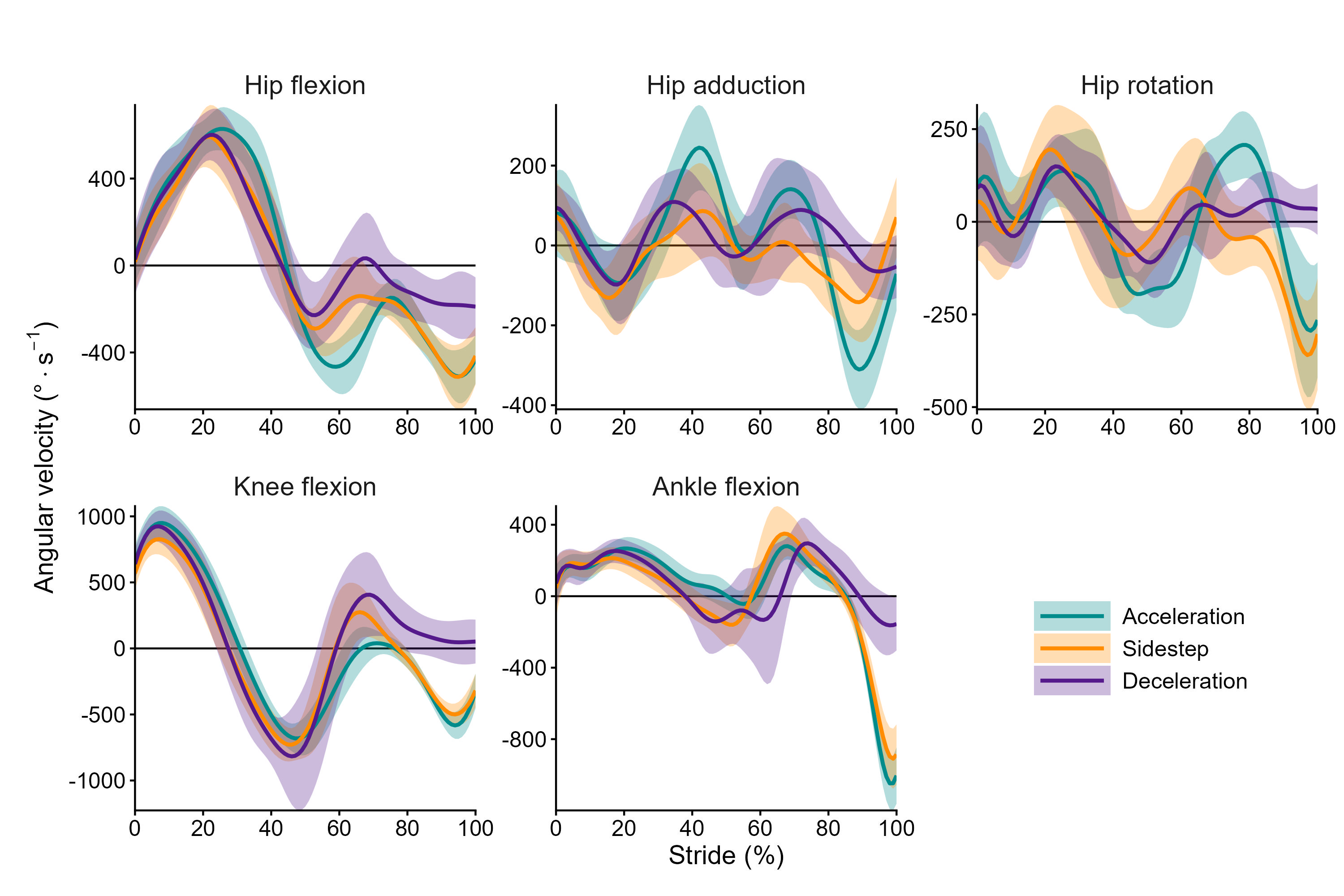


Supplementary Figure S4. Mean (line) and SD (shaded area) joint angular velocities for the stride cycle (toe-off to toe-off) of acceleration (green), 45-degree sidestep cutting (orange) and deceleration (purple). Note that the stride cycle corresponds to the final foot contact prior to change of direction (for sidestep cutting) and the first decelerative step (for deceleration). Positive values indicate hip flexion, hip adduction, hip internal rotation, knee flexion and ankle dorsi flexion, for each subplot.


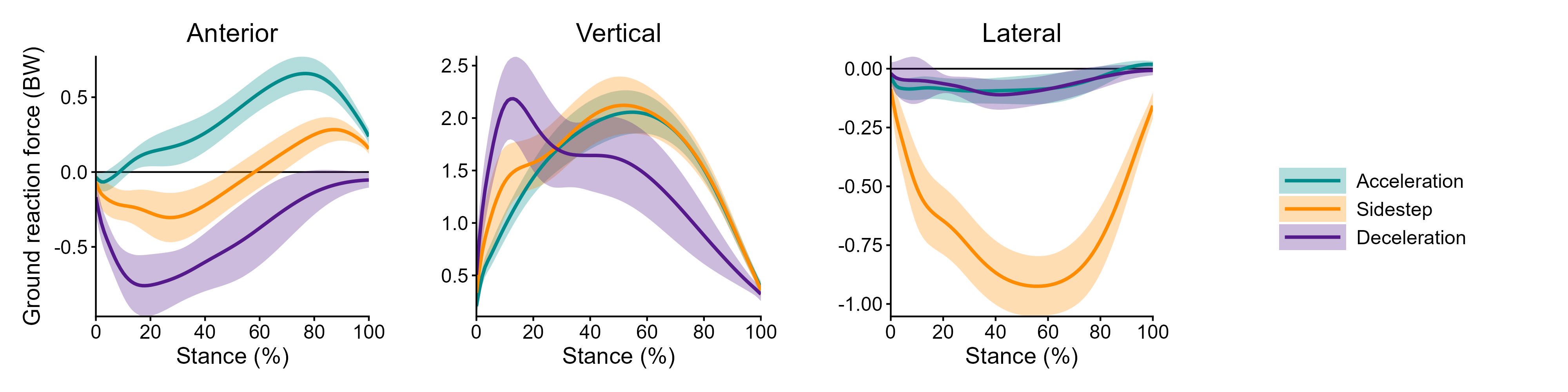


Supplementary Figure S5. Mean (line) and SD (shaded area) ground reaction forces for the stance phase of acceleration (green), 45-degree sidestep cutting (orange) and deceleration (purple). Note that the stance phase corresponds to the final foot contact prior to change of direction (for sidestep cutting) and the first decelerative step (for deceleration). Positive values indicate anterior, vertical, and medial (i.e., toward the left for right-foot contact), for each subplot.


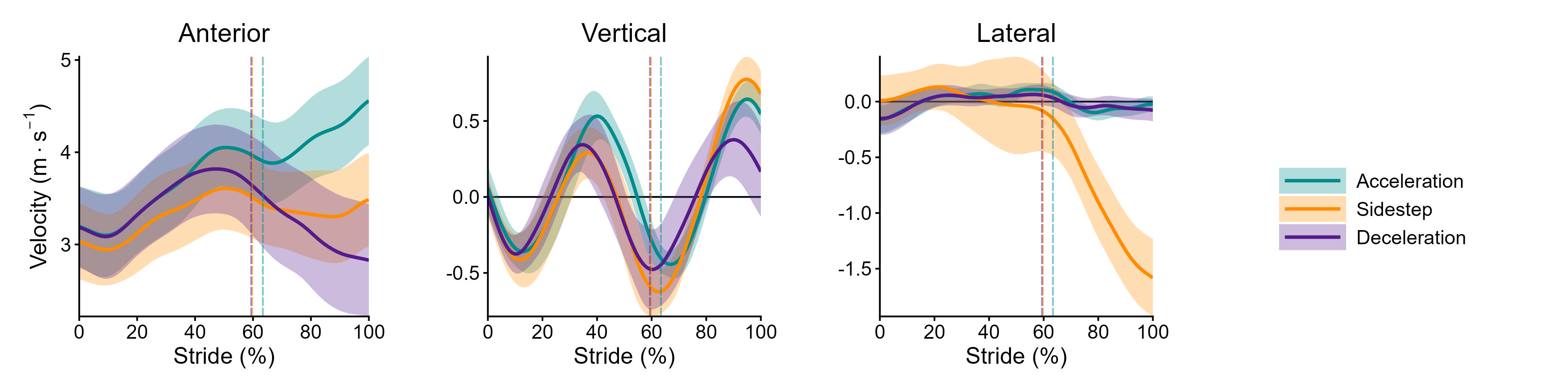


Supplementary Figure S6. Mean (line) and SD (shaded area) centre of mass velocity for the stride cycle of acceleration (green), 45-degree sidestep cutting (orange) and deceleration (purple). Note that the stride cycle corresponds to the final foot contact prior to change of direction (for sidestep cutting) and the first decelerative step (for deceleration). Positive values indicate anterior, vertical, and medial (i.e., toward the left for right-foot contact), for each subplot. The vertical dashed lines indicate the beginning of the stance phase for each task.

Supplementary Material S4: Comparison of experimental and model-derived data

This supplement contains a comparison of model-derived estimates against experimental data to validate and verify our simulations. Specifically, we comparisons are provided for:

- normalised electromyography (EMG) and the model-derived muscle force estimates for each task
- body mass normalised joint moments for inverse dynamics and the model-derived muscle moment for each task

For EMG and muscle force comparisons, outcomes were normalised to the peak value obtained across all trials/tasks, to facilitate comparison. Note also that this comparison was run for n=18 participants, owing to technical failures for EMG data collection for two participants.


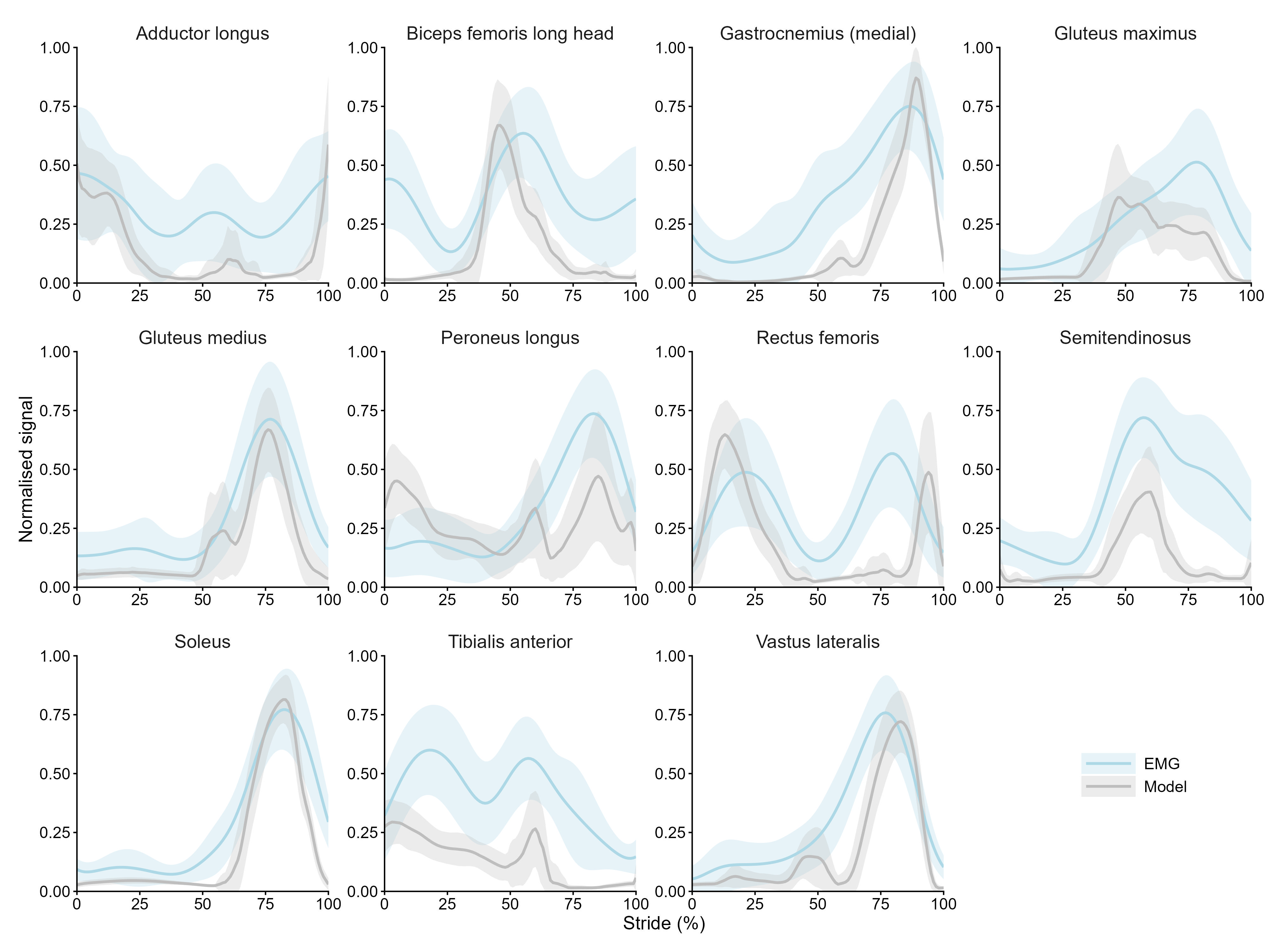


Supplementary Figure S7. Mean (line) and SD (shaded area) normalised electromyography (EMG, blue) and model-derived muscle force (grey) for the stride cycle (toe-off to toe-off) for acceleration. Note that normalisation was performed across all trials (including sidestep cutting and deceleration).


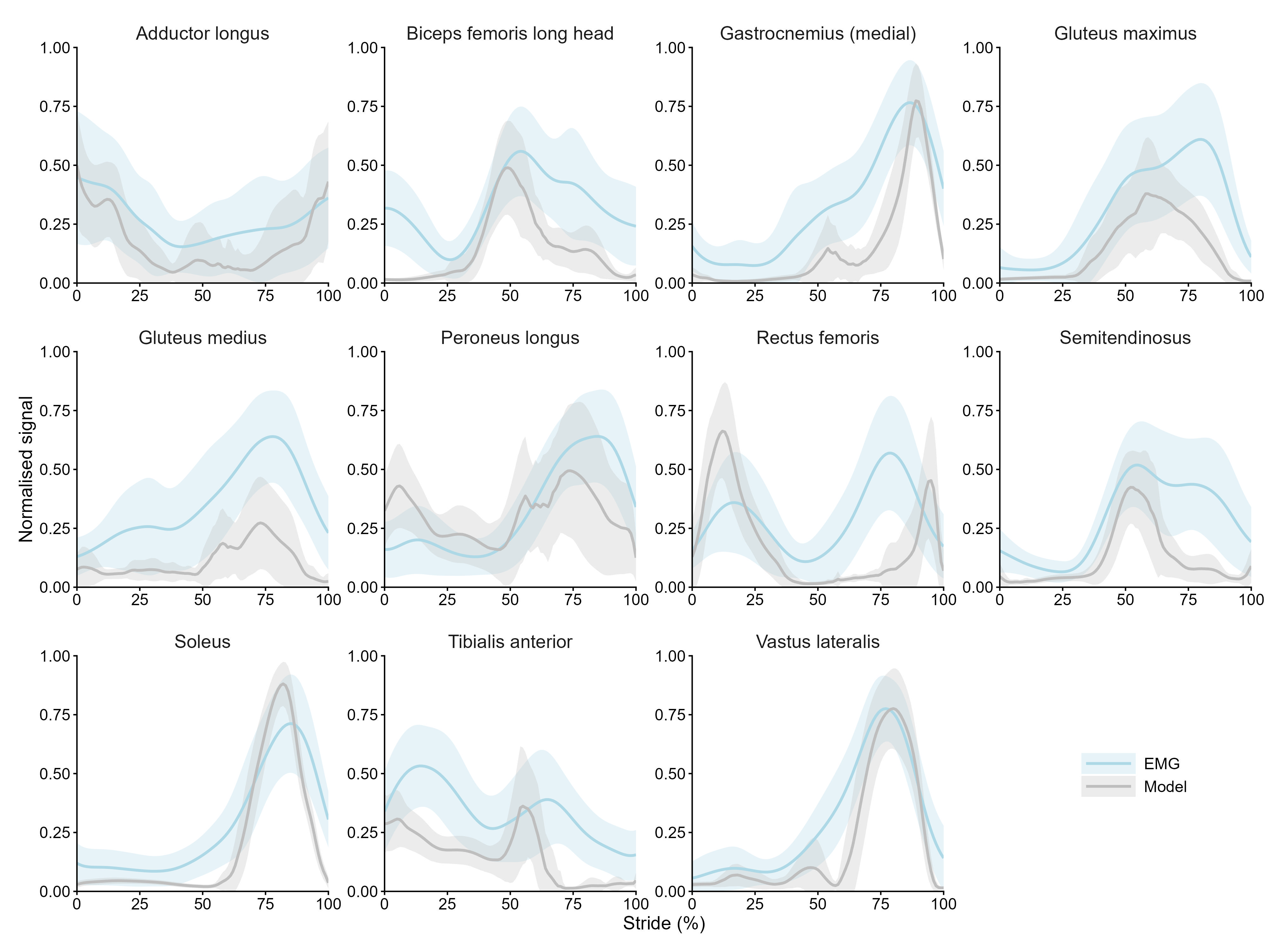


Supplementary Figure S8. Mean (line) and SD (shaded area) normalised electromyography (EMG, blue) and model-derived muscle force (grey) for the stride cycle (toe-off to toe-off) for 45-degree sidestep cutting. Note that normalisation was performed across all trials (including acceleration and deceleration).


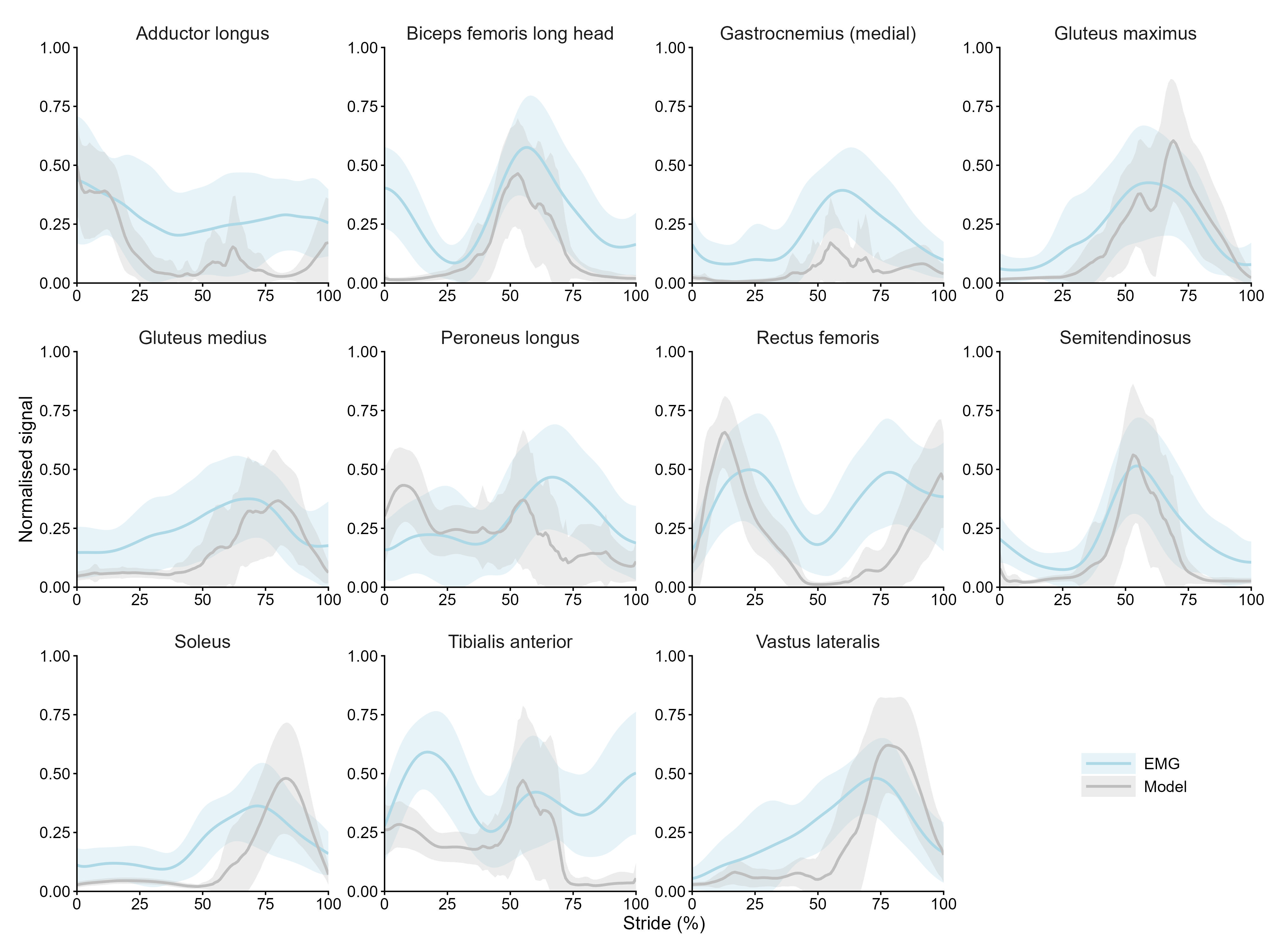


Supplementary Figure S9. Mean (line) and SD (shaded area) normalised electromyography (EMG, blue) and model-derived muscle force (grey) for the stride cycle (toe-off to toe-off) for deceleration. Note that normalisation was performed across all trials (including sidestep cutting and acceleration).


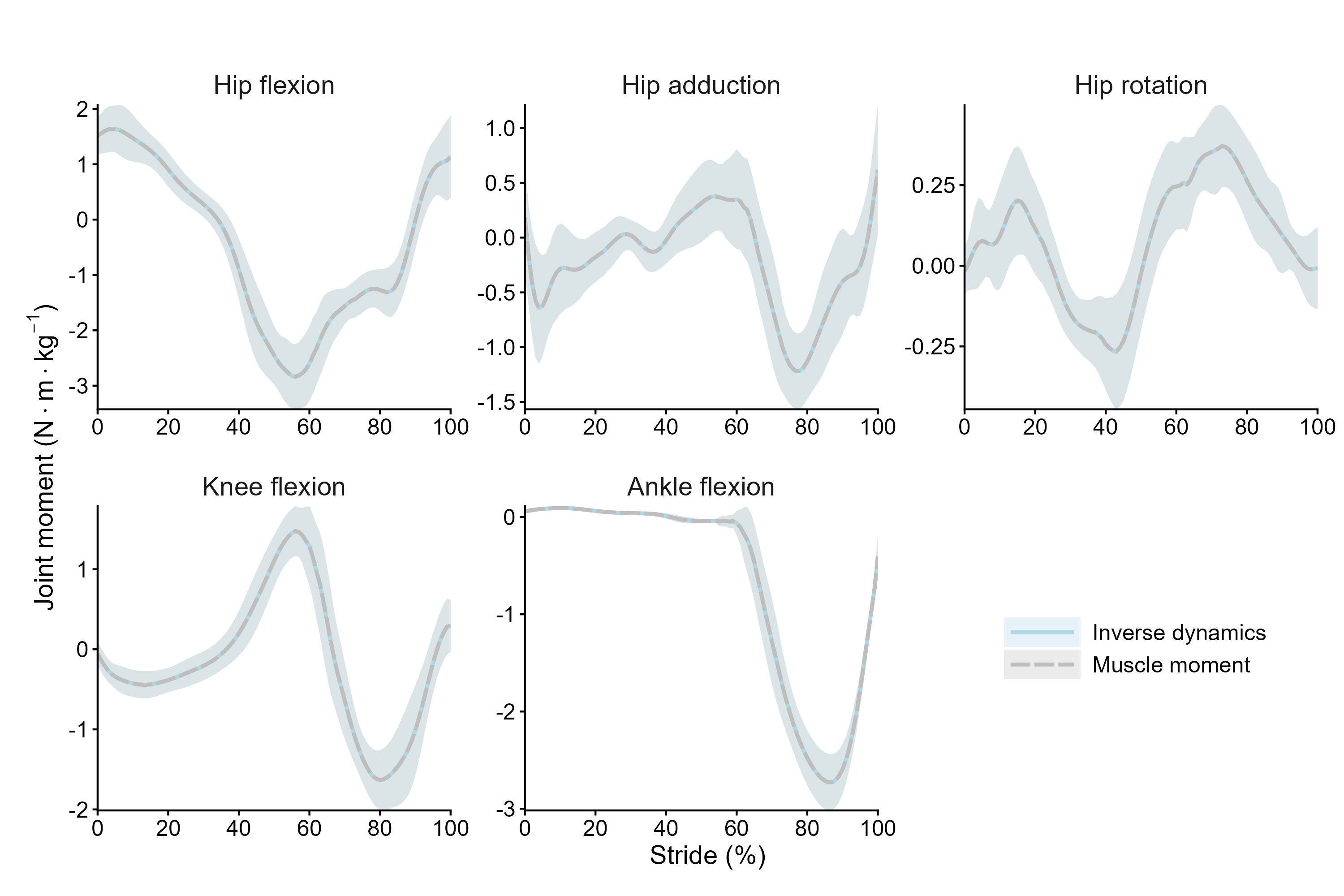


Supplementary Figure S10. Mean (line) and SD (shaded area) body-mass normalised joint moments from inverse dynamics (blue) and model-derived muscle moment (grey) for the stride cycle (toe-off to toe-off) for acceleration.


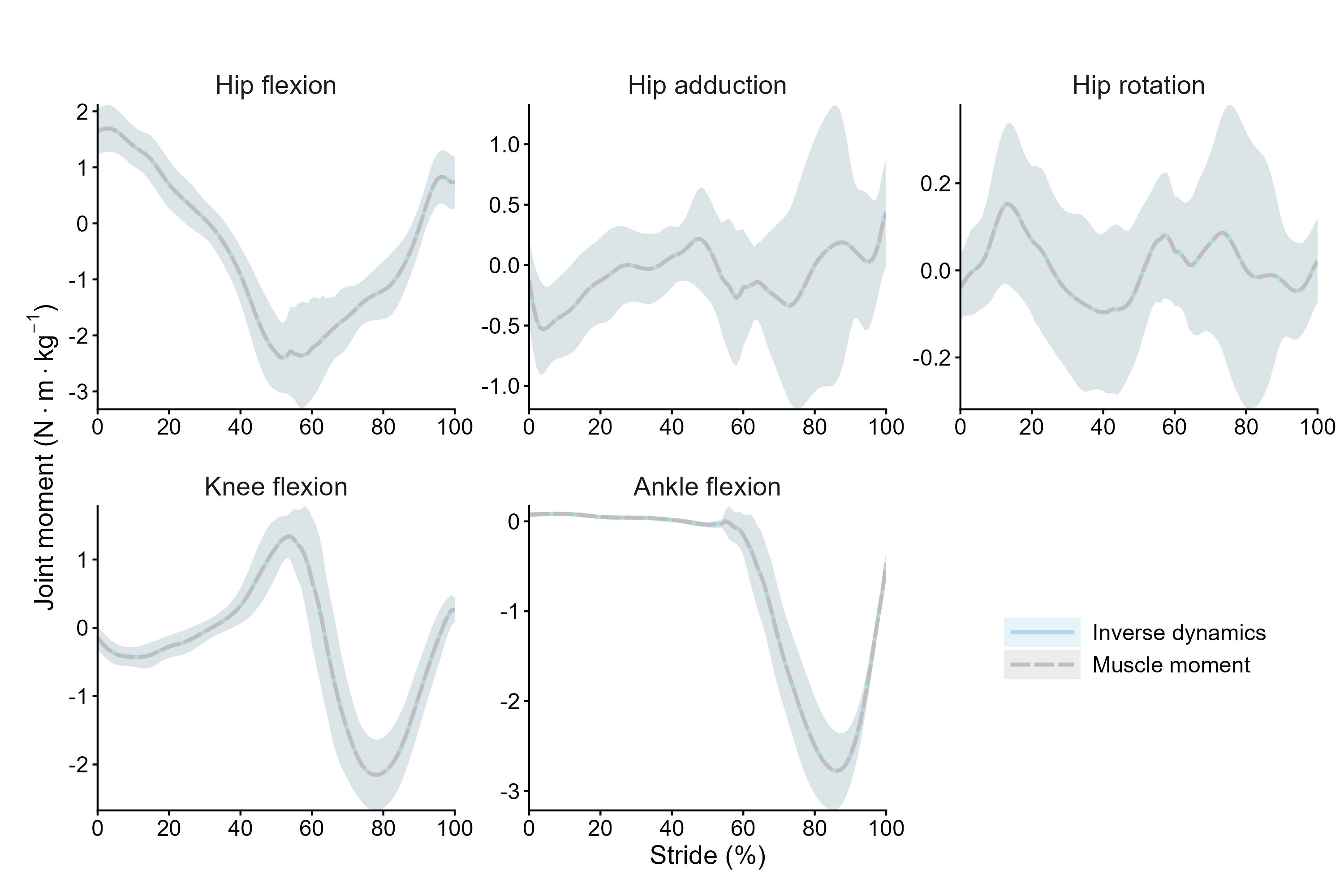


Supplementary Figure S11. Mean (line) and SD (shaded area) body-mass normalised joint moments from inverse dynamics (blue) and model-derived muscle moment (grey) for the stride cycle (toe-off to toe-off) for 45-degree sidestep cutting.


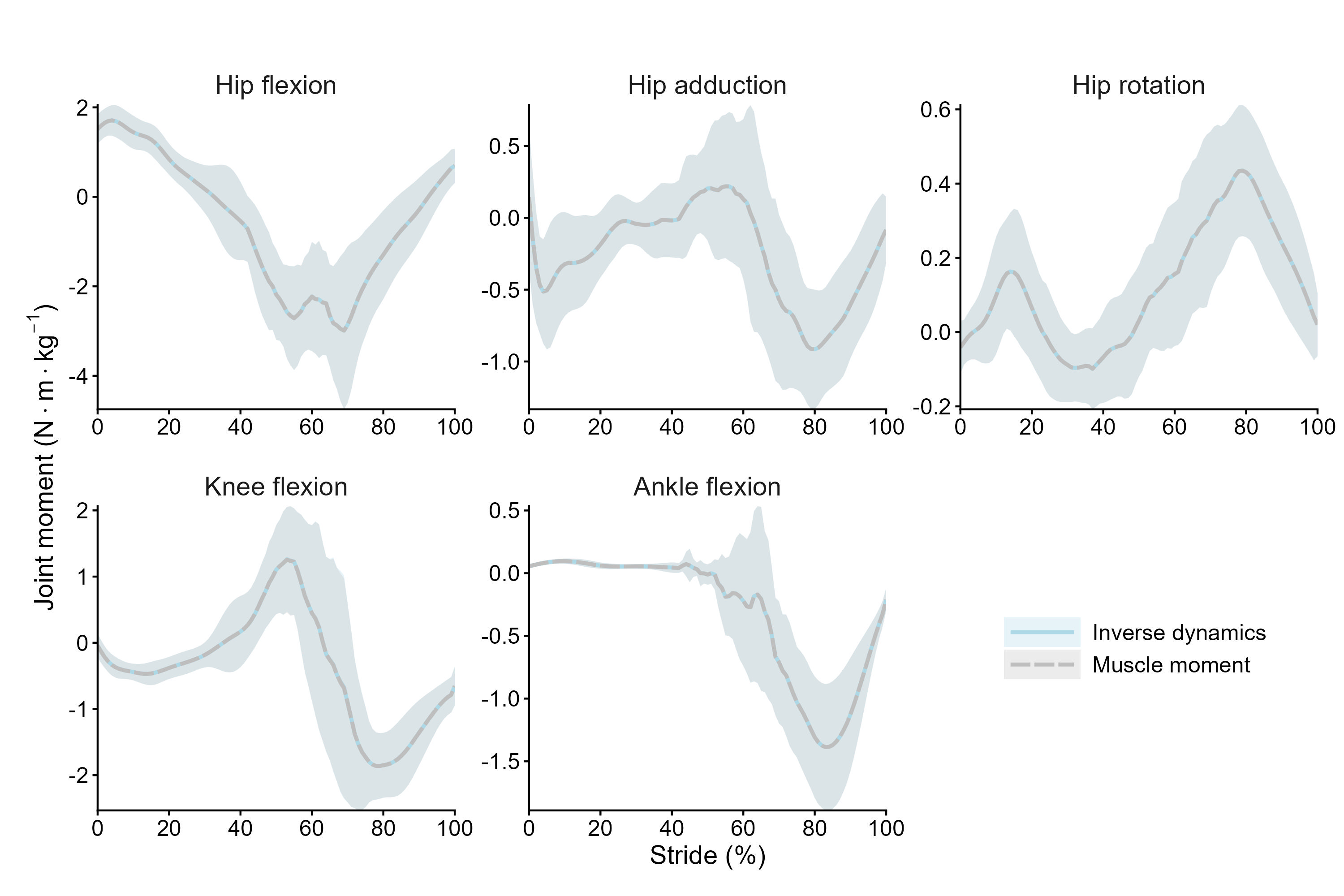


Supplementary Figure S12. Mean (line) and SD (shaded area) body-mass normalised joint moments from inverse dynamics (blue) and model-derived muscle moment (grey) for the stride cycle (toe-off to toe-off) for deceleration.

Supplementary Material S5: Individual variability curves

This supplement demonstrates the individual variability of our key outcome variables related to the biarticular hamstrings:

- force
- power
- stretch
- velocity


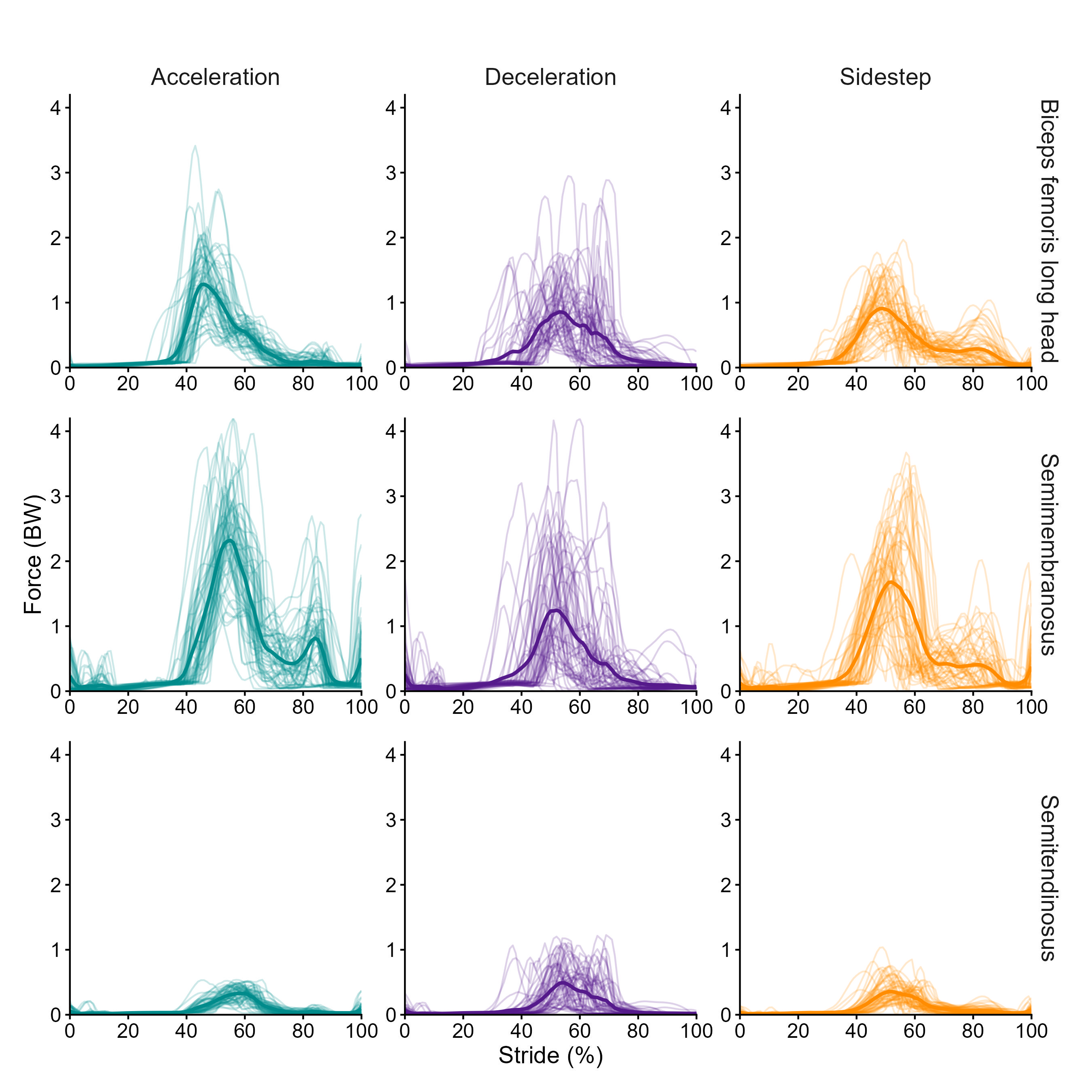


Supplementary Figure S13. Mean (thick line) and individual (faded lines) of the bodyweight (BW) normalised musculotendinous forces for the biceps femoris long head (top row), semimembranosus (second row) and semitendinosus (bottom row) for the stride cycle (toe-off to toe-eff) of acceleration (green, first column), deceleration (purple, second column), and 45-degree sidestep cutting (orange, third column). Note that the stride cycle corresponds to the final foot contact prior to change of direction (for sidestep cutting) and the first decelerative step (for deceleration).


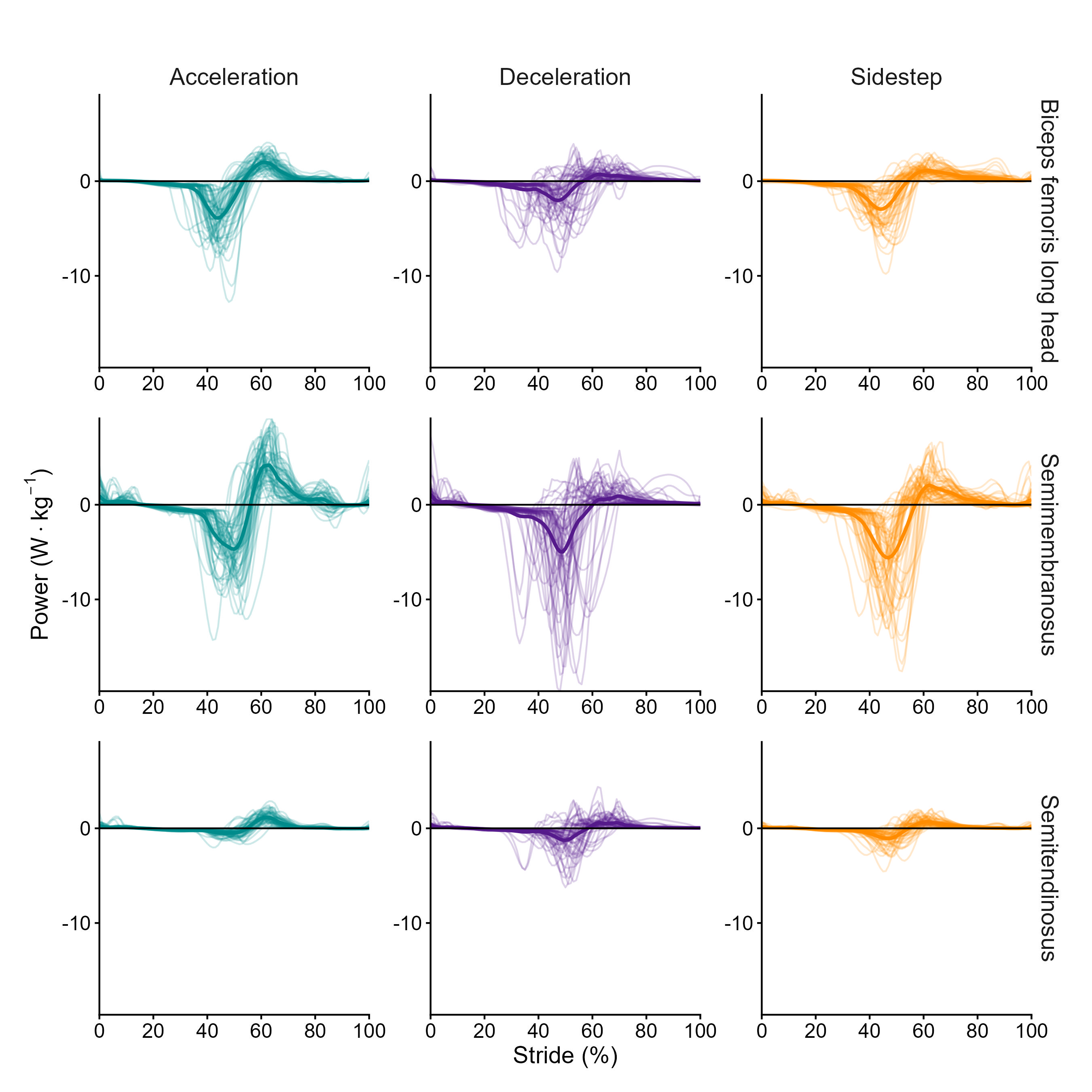


Supplementary Figure S14. Mean (thick line) and individual (faded lines) of the body mass normalised musculotendinous for the biceps femoris long head (top row), semimembranosus (second row) and semitendinosus (bottom row) for the stride cycle (toe-off to toe-eff) of acceleration (green, first column), deceleration (purple, second column), and 45-degree sidestep cutting (orange, third column). Note that the stride cycle corresponds to the final foot contact prior to change of direction (for sidestep cutting) and the first decelerative step (for deceleration). Positive values indicate power generation.


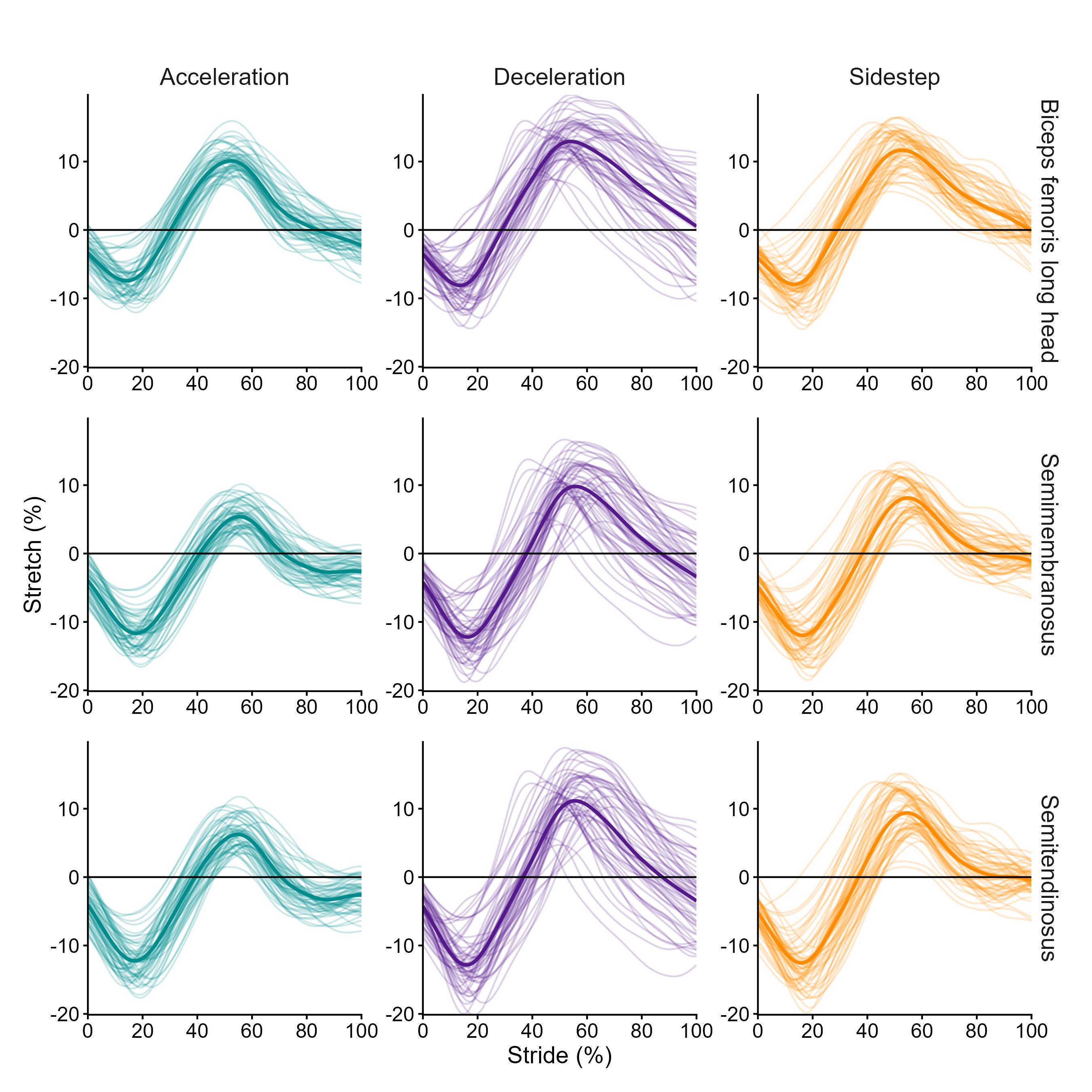


Supplementary Figure S15. Mean (thick line) and individual (faded lines) of the musculotendinous (MTU) stretch for the biceps femoris long head (top row), semimembranosus (second row) and semitendinosus (bottom row) for the stride cycle (toe-off to toe-eff) of acceleration (green, first column), deceleration (purple, second column), and 45-degree sidestep cutting (orange, third column). Note that the stride cycle corresponds to the final foot contact prior to change of direction (for sidestep cutting) and the first decelerative step (for deceleration). Positive values indicate MTU lengths greater than anatomical position.


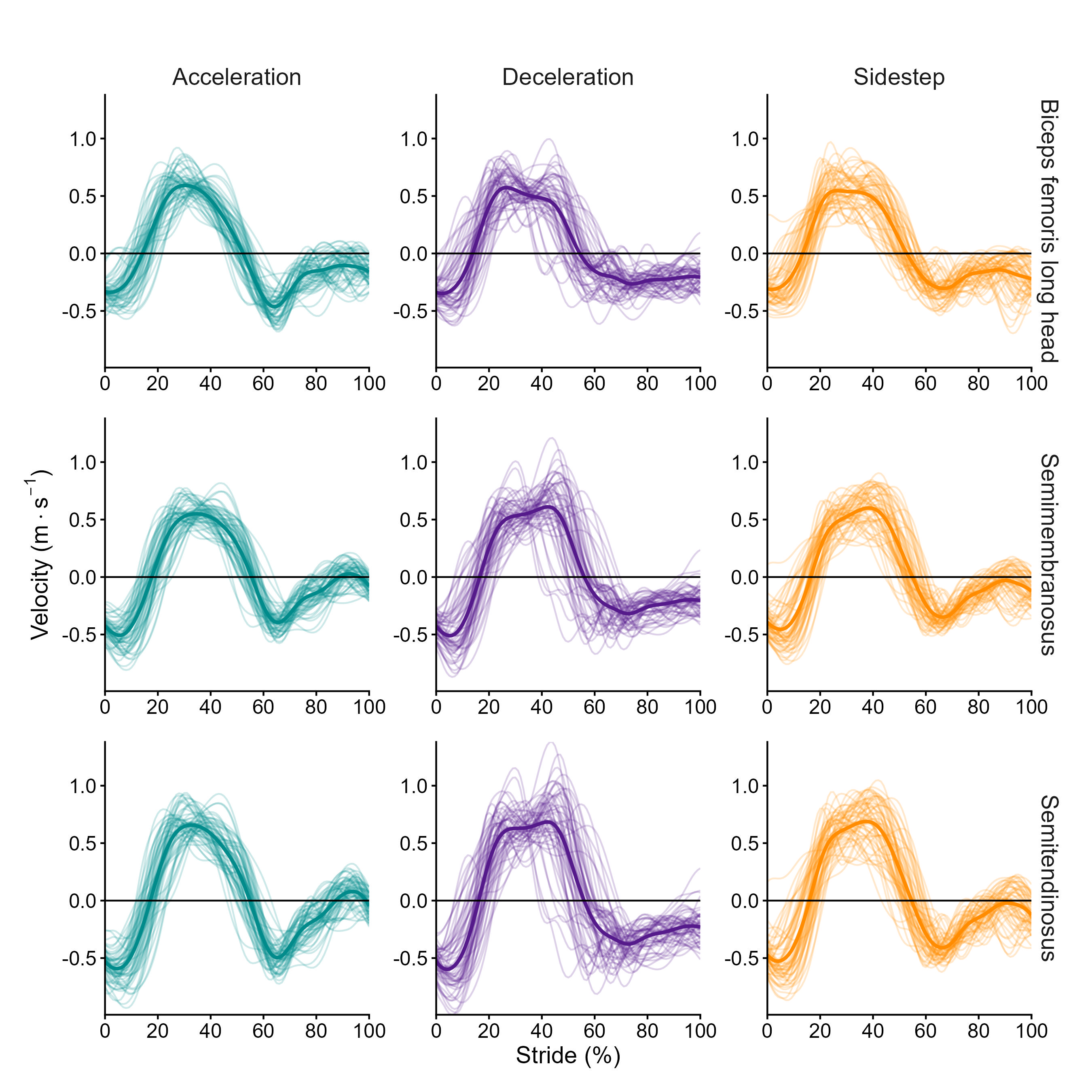


Supplementary Figure S16. Mean (thick line) and individual (faded lines) of the musculotendinous (MTU) velocity for the biceps femoris long head (top row), semimembranosus (second row) and semitendinosus (bottom row) for the stride cycle (toe-off to toe-eff) of acceleration (green, first column), deceleration (purple, second column), and 45-degree sidestep cutting (orange, third column). Note that the stride cycle corresponds to the final foot contact prior to change of direction (for sidestep cutting) and the first decelerative step (for deceleration). Positive values indicate MTU lengthening.

Supplementary Material S6: Missing data

This supplementary data contains a visualisation of our missing data. Since the total missing data was small (5%) and deemed missing completely at random, no adjustment to our statistical analysis was performed (e.g., multiple imputation). Specific reasons for missing data include:

- Deceleration trials not truly decelerative (n = 2)
- Data artefacts (n = 7)


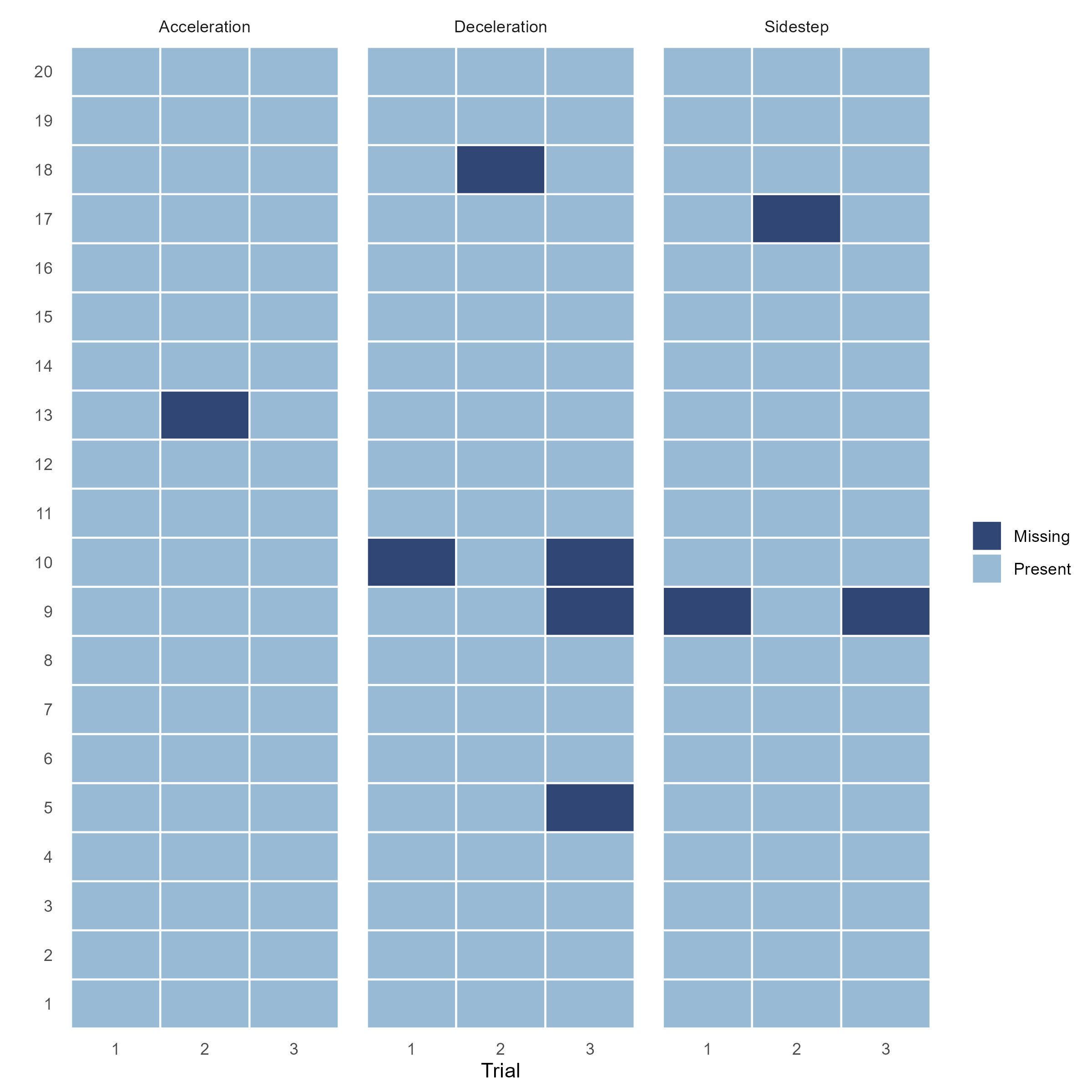


Supplementary Figure S17. Visualisation of missing (dark blue) and present (light blue) data for each trial (x-axis) and participant (y-axis) for data included in our statistical analysis and summary figures. Note that electromyography data had additional missing data (technical failures for two participants), but this was not included in this visualisation as it was not related to our key outcomes.

Supplementary Material S7: Statistical data

This supplementary data contains statistical information supplementary to our manuscript. This includes:

- Marginal means and 95%CI for stance phase outcome variables (peak force, peak power generation, and positive work)
- Pairwise contrasts for all linear mixed models of the swing phase
- Pairwise contrasts for all linear mixed models of the stance phase

Supplementary Table S2: Marginal means and 95%CI confidence interval for secondary outcomes in the stance phase of acceleration, deceleration and sidestep cutting.

|  | Marginal mean and 95%CI | | |
| --- | --- | --- | --- |
| Muscle | Acceleration | Cut | Deceleration |
| Force (BW) | | | |
| Biceps femoris long head | 0.42 [0.29 to 0.55] | 0.66 [0.52 to 0.79] | 1.23 [1.10 to 1.36] |
| Semimembranosus | 1.39 [1.17 to 1.61] | 1.27 [1.04 to 1.49] | 1.14 [0.92 to 1.36] |
| Semitendinosus | 0.24 [0.19 to 0.30] | 0.35 [0.29 to 0.40] | 0.61 [0.56 to 0.67] |
| Power generation (W⋅kg^-1^) | | | |
| Biceps femoris long head | 1.83 [1.55 to 2.11] | 1.59 [1.30 to 1.88] | 1.62 [1.34 to 1.90] |
| Semimembranosus | 4.18 [3.57 to 4.79] | 2.84 [2.21 to 3.47] | 2.34 [1.74 to 2.95] |
| Semitendinosus | 1.10 [0.90 to 1.29] | 1.09 [0.88 to 1.29] | 1.53 [1.34 to 1.73] |
| Positive work (J⋅kg^-1^) | | | |
| Biceps femoris long head | 0.05 [0.04 to 0.06] | 0.11 [0.09 to 0.12] | 0.08 [0.06 to 0.09] |
| Semimembranosus | 0.14 [0.11 to 0.16] | 0.15 [0.13 to 0.18] | 0.11 [0.09 to 0.14] |
| Semitendinosus | 0.03 [0.02 to 0.03] | 0.05 [0.04 to 0.05] | 0.06 [0.05 to 0.07] |
| Marginal estimates were adjusted to the mean approach velocity (3.48 m⋅s^-1^).  Abbreviations: CI, confidence interval; BW, bodyweight. | | | |

Supplementary Table S3: Pairwise contrasts for linear mixed models of the swing phase for acceleration, deceleration and sidestep cutting.

| Muscle | Contrast | Mean difference [95%CI] | Standardised difference [95%CI] | df | p |
| --- | --- | --- | --- | --- | --- |
| Force (BW) | | | | | |
| Biceps femoris long head | acceleration - sidestep | 0.26 [0.11 to 0.42] | 0.55 [0.22 to 0.88] | 162.62 | <0.001 |
| Biceps femoris long head | acceleration - deceleration | 0.25 [0.12 to 0.38] | 0.52 [0.25 to 0.79] | 154.09 | <0.001 |
| Biceps femoris long head | sidestep - deceleration | -0.01 [-0.16 to 0.13] | -0.03 [-0.33 to 0.27] | 166.49 | 0.970 |
| Semimembranosus | acceleration - sidestep | 0.37 [0.09 to 0.65] | 0.43 [0.10 to 0.75] | 162.29 | 0.007 |
| Semimembranosus | acceleration - deceleration | 0.77 [0.53 to 1.01] | 0.89 [0.61 to 1.16] | 154.13 | <0.001 |
| Semimembranosus | sidestep - deceleration | 0.40 [0.14 to 0.66] | 0.46 [0.16 to 0.76] | 166.53 | 0.001 |
| Semitendinosus | acceleration - sidestep | -0.13 [-0.20 to -0.06] | -0.57 [-0.88 to -0.25] | 162.25 | <0.001 |
| Semitendinosus | acceleration - deceleration | -0.33 [-0.39 to -0.27] | -1.47 [-1.75 to -1.19] | 153.96 | <0.001 |
| Semitendinosus | sidestep - deceleration | -0.20 [-0.27 to -0.13] | -0.90 [-1.20 to -0.60] | 166.28 | <0.001 |
| Power absorption (W⋅kg^-1^) | | | | | |
| Biceps femoris long head | acceleration - sidestep | -0.98 [-1.74 to -0.22] | -0.43 [-0.76 to -0.10] | 163.15 | 0.007 |
| Biceps femoris long head | acceleration - deceleration | -1.26 [-1.89 to -0.63] | -0.55 [-0.83 to -0.28] | 154.01 | <0.001 |
| Biceps femoris long head | sidestep - deceleration | -0.28 [-0.98 to 0.42] | -0.12 [-0.43 to 0.18] | 166.40 | 0.613 |
| Semimembranosus | acceleration - sidestep | 2.64 [1.16 to 4.13] | 0.65 [0.29 to 1.02] | 161.56 | <0.001 |
| Semimembranosus | acceleration - deceleration | 2.66 [1.35 to 3.97] | 0.66 [0.33 to 0.98] | 154.08 | <0.001 |
| Semimembranosus | sidestep - deceleration | 0.01 [-1.40 to 1.43] | 0.00 [-0.34 to 0.35] | 166.46 | 1.000 |
| Semitendinosus | acceleration - sidestep | 0.94 [0.53 to 1.36] | 0.77 [0.43 to 1.10] | 162.18 | <0.001 |
| Semitendinosus | acceleration - deceleration | 1.65 [1.28 to 2.02] | 1.34 [1.04 to 1.64] | 153.97 | <0.001 |
| Semitendinosus | sidestep - deceleration | 0.71 [0.31 to 1.10] | 0.57 [0.25 to 0.90] | 166.30 | <0.001 |
| Negative work (J⋅kg^-1^) | | | | | |
| Biceps femoris long head | acceleration - sidestep | 0.00 [-0.04 to 0.04] | 0.00 [-0.34 to 0.35] | 162.80 | 1.000 |
| Biceps femoris long head | acceleration - deceleration | -0.04 [-0.07 to -0.01] | -0.36 [-0.64 to -0.07] | 154.06 | 0.010 |
| Biceps femoris long head | sidestep - deceleration | -0.04 [-0.07 to 0.00] | -0.36 [-0.68 to -0.05] | 166.46 | 0.021 |
| Semimembranosus | acceleration - sidestep | 0.17 [0.11 to 0.24] | 0.94 [0.59 to 1.30] | 162.59 | <0.001 |
| Semimembranosus | acceleration - deceleration | 0.10 [0.04 to 0.15] | 0.52 [0.20 to 0.84] | 153.90 | 0.001 |
| Semimembranosus | sidestep - deceleration | -0.08 [-0.14 to -0.02] | -0.42 [-0.77 to -0.08] | 166.18 | 0.010 |
| Semitendinosus | acceleration - sidestep | 0.05 [0.03 to 0.07] | 0.99 [0.66 to 1.32] | 162.41 | <0.001 |
| Semitendinosus | acceleration - deceleration | 0.07 [0.06 to 0.09] | 1.42 [1.12 to 1.71] | 153.93 | <0.001 |
| Semitendinosus | sidestep - deceleration | 0.02 [0.01 to 0.04] | 0.43 [0.11 to 0.74] | 166.23 | 0.005 |
| Stretch (%) | | | | | |
| Biceps femoris long head | acceleration - sidestep | -2.25 [-3.05 to -1.46] | -0.80 [-1.08 to -0.52] | 165.23 | <0.001 |
| Biceps femoris long head | acceleration - deceleration | -3.55 [-4.20 to -2.91] | -1.26 [-1.48 to -1.03] | 153.60 | <0.001 |
| Biceps femoris long head | sidestep - deceleration | -1.30 [-2.02 to -0.57] | -0.46 [-0.72 to -0.20] | 165.82 | <0.001 |
| Semimembranosus | acceleration - sidestep | -3.32 [-4.21 to -2.42] | -0.98 [-1.24 to -0.72] | 164.33 | <0.001 |
| Semimembranosus | acceleration - deceleration | -5.40 [-6.13 to -4.67] | -1.60 [-1.81 to -1.38] | 153.81 | <0.001 |
| Semimembranosus | sidestep - deceleration | -2.08 [-2.90 to -1.26] | -0.62 [-0.86 to -0.37] | 166.13 | <0.001 |
| Semitendinosus | acceleration - sidestep | -3.77 [-4.78 to -2.76] | -1.00 [-1.26 to -0.73] | 164.14 | <0.001 |
| Semitendinosus | acceleration - deceleration | -5.96 [-6.79 to -5.13] | -1.57 [-1.79 to -1.36] | 153.85 | <0.001 |
| Semitendinosus | sidestep - deceleration | -2.19 [-3.12 to -1.26] | -0.58 [-0.82 to -0.33] | 166.18 | <0.001 |
| Lengthening velocity (m⋅s^-1^) | | | | | |
| Biceps femoris long head | acceleration - sidestep | 0.00 [-0.05 to 0.04] | -0.02 [-0.39 to 0.35] | 162.47 | 0.992 |
| Biceps femoris long head | acceleration - deceleration | -0.03 [-0.07 to 0.01] | -0.25 [-0.56 to 0.05] | 154.11 | 0.129 |
| Biceps femoris long head | sidestep - deceleration | -0.03 [-0.07 to 0.01] | -0.23 [-0.57 to 0.11] | 166.51 | 0.236 |
| Semimembranosus | acceleration - sidestep | -0.07 [-0.12 to -0.02] | -0.49 [-0.87 to -0.12] | 162.52 | 0.007 |
| Semimembranosus | acceleration - deceleration | -0.15 [-0.20 to -0.10] | -1.11 [-1.45 to -0.78] | 153.91 | <0.001 |
| Semimembranosus | sidestep - deceleration | -0.08 [-0.13 to -0.04] | -0.62 [-0.98 to -0.26] | 166.20 | <0.001 |
| Semitendinosus | acceleration - sidestep | -0.06 [-0.12 to 0.00] | -0.40 [-0.79 to -0.02] | 161.90 | 0.037 |
| Semitendinosus | acceleration - deceleration | -0.14 [-0.19 to -0.09] | -0.93 [-1.27 to -0.59] | 154.02 | <0.001 |
| Semitendinosus | sidestep - deceleration | -0.08 [-0.13 to -0.02] | -0.53 [-0.89 to -0.16] | 166.37 | 0.003 |
| Marginal estimates were adjusted to the mean approach velocity (3.48 m⋅s^-1^). | | | | | |

Supplementary Table S4: Pairwise contrasts for linear mixed models of the stance phase for acceleration, deceleration and sidestep cutting.

| Muscle | Contrast | Mean difference [95%CI] | Standardised difference [95%CI] | df | p |
| --- | --- | --- | --- | --- | --- |
| Force (BW) | | | | | |
| Biceps femoris long head | acceleration - sidestep | -0.24 [-0.40 to -0.07] | -0.45 [-0.78 to -0.13] | 163.78 | 0.003 |
| Biceps femoris long head | acceleration - deceleration | -0.81 [-0.96 to -0.66] | -1.56 [-1.86 to -1.27] | 153.70 | <0.001 |
| Biceps femoris long head | sidestep - deceleration | -0.58 [-0.74 to -0.41] | -1.11 [-1.42 to -0.80] | 165.78 | <0.001 |
| Semimembranosus | acceleration - sidestep | 0.12 [-0.13 to 0.38] | 0.19 [-0.21 to 0.59] | 161.70 | 0.496 |
| Semimembranosus | acceleration - deceleration | 0.24 [0.02 to 0.47] | 0.38 [0.03 to 0.73] | 154.06 | 0.030 |
| Semimembranosus | sidestep - deceleration | 0.12 [-0.12 to 0.36] | 0.19 [-0.19 to 0.57] | 166.43 | 0.464 |
| Semitendinosus | acceleration - sidestep | -0.10 [-0.18 to -0.03] | -0.43 [-0.75 to -0.11] | 164.84 | 0.005 |
| Semitendinosus | acceleration - deceleration | -0.37 [-0.44 to -0.30] | -1.55 [-1.84 to -1.26] | 153.52 | <0.001 |
| Semitendinosus | sidestep - deceleration | -0.27 [-0.34 to -0.19] | -1.12 [-1.43 to -0.82] | 165.32 | <0.001 |
| Power generation (W⋅kg^-1^) | | | | | |
| Biceps femoris long head | acceleration - sidestep | 0.24 [-0.05 to 0.53] | 0.31 [-0.06 to 0.68] | 161.29 | 0.117 |
| Biceps femoris long head | acceleration - deceleration | 0.21 [-0.04 to 0.46] | 0.27 [-0.05 to 0.59] | 154.21 | 0.109 |
| Biceps femoris long head | sidestep - deceleration | -0.03 [-0.30 to 0.24] | -0.04 [-0.39 to 0.30] | 166.62 | 0.959 |
| Semimembranosus | acceleration - sidestep | 1.34 [0.76 to 1.91] | 0.75 [0.43 to 1.08] | 162.09 | <0.001 |
| Semimembranosus | acceleration - deceleration | 1.84 [1.35 to 2.32] | 1.04 [0.76 to 1.31] | 154.15 | <0.001 |
| Semimembranosus | sidestep - deceleration | 0.50 [-0.03 to 1.03] | 0.28 [-0.02 to 0.58] | 166.56 | 0.072 |
| Semitendinosus | acceleration - sidestep | 0.01 [-0.25 to 0.27] | 0.02 [-0.38 to 0.41] | 164.01 | 0.995 |
| Semitendinosus | acceleration - deceleration | -0.44 [-0.67 to -0.20] | -0.67 [-1.03 to -0.31] | 153.66 | <0.001 |
| Semitendinosus | sidestep - deceleration | -0.45 [-0.70 to -0.20] | -0.69 [-1.07 to -0.31] | 165.69 | <0.001 |
| Positive work (J⋅kg^-1^) | | | | | |
| Biceps femoris long head | acceleration - sidestep | -0.06 [-0.07 to -0.04] | -1.35 [-1.70 to -0.99] | 163.43 | <0.001 |
| Biceps femoris long head | acceleration - deceleration | -0.03 [-0.04 to -0.01] | -0.62 [-0.94 to -0.30] | 153.76 | <0.001 |
| Biceps femoris long head | sidestep - deceleration | 0.03 [0.02 to 0.05] | 0.72 [0.38 to 1.07] | 165.91 | <0.001 |
| Semimembranosus | acceleration - sidestep | -0.02 [-0.04 to 0.01] | -0.28 [-0.66 to 0.10] | 161.19 | 0.191 |
| Semimembranosus | acceleration - deceleration | 0.02 [0.00 to 0.04] | 0.34 [0.01 to 0.66] | 154.21 | 0.041 |
| Semimembranosus | sidestep - deceleration | 0.04 [0.02 to 0.06] | 0.62 [0.26 to 0.97] | 166.61 | <0.001 |
| Semitendinosus | acceleration - sidestep | -0.02 [-0.03 to -0.01] | -0.82 [-1.17 to -0.47] | 164.28 | <0.001 |
| Semitendinosus | acceleration - deceleration | -0.03 [-0.04 to -0.03] | -1.35 [-1.67 to -1.04] | 153.61 | <0.001 |
| Semitendinosus | sidestep - deceleration | -0.01 [-0.02 to 0.00] | -0.54 [-0.87 to -0.20] | 165.58 | 0.001 |
| Marginal estimates were adjusted to the mean approach velocity (3.48 m⋅s^-1^). | | | | | |
